# The genome and transcriptome of perennial ryegrass mitochondria

**DOI:** 10.1186/1471-2164-14-202

**Published:** 2013-03-23

**Authors:** Md Shofiqul Islam, Bruno Studer, Stephen L Byrne, Jacqueline D Farrell, Frank Panitz, Christian Bendixen, Ian Max Møller, Torben Asp

**Affiliations:** 1 Department of Molecular Biology and Genetics, Science and Technology, Aarhus University, Forsøgsvej 1, Slagelse, DK 4200, Denmark; 2 Department of Environmental Systems Science, Forage Crop Genetics, ETH Zurich, Zurich, CH 8092, Switzerland; 3 Department of Molecular Biology and Genetics, Science and Technology, Aarhus University, Blichers Allé 20, Tjele, DK 8830, Denmark

**Keywords:** *De novo* assembly, Mitochondrial gene expression, Mitochondrial genome, Next-generation sequencing, Perennial ryegrass (*Lolium perenne* L.)

## Abstract

**Background:**

Perennial ryegrass (*Lolium perenne* L.) is one of the most important forage and turf grass species of temperate regions worldwide. Its mitochondrial genome is inherited maternally and contains genes that can influence traits of agricultural importance. Moreover, the DNA sequence of mitochondrial genomes has been established and compared for a large number of species in order to characterize evolutionary relationships. Therefore, it is crucial to understand the organization of the mitochondrial genome and how it varies between and within species. Here, we report the first *de novo* assembly and annotation of the complete mitochondrial genome from perennial ryegrass.

**Results:**

Intact mitochondria from perennial ryegrass leaves were isolated and used for mtDNA extraction. The mitochondrial genome was sequenced to a 167-fold coverage using the Roche 454 GS-FLX Titanium platform, and assembled into a circular master molecule of 678,580 bp. A total of 34 proteins, 14 tRNAs and 3 rRNAs are encoded by the mitochondrial genome, giving a total gene space of 48,723 bp (7.2%). Moreover, we identified 149 open reading frames larger than 300 bp and covering 67,410 bp (9.93%), 250 SSRs, 29 tandem repeats, 5 pairs of large repeats, and 96 pairs of short inverted repeats. The genes encoding subunits of the respiratory complexes – *nad*1 to *nad*9, *cob*, *cox*1 to *cox*3 and *atp*1 to *atp*9 – all showed high expression levels both in absolute numbers and after normalization.

**Conclusions:**

The circular master molecule of the mitochondrial genome from perennial ryegrass presented here constitutes an important tool for future attempts to compare mitochondrial genomes within and between grass species. Our results also demonstrate that mitochondria of perennial ryegrass contain genes crucial for energy production that are well conserved in the mitochondrial genome of monocotyledonous species. The expression analysis gave us first insights into the transcriptome of these mitochondrial genes in perennial ryegrass.

## Background

Mitochondria are semi-autonomous organelles in eukaryotes. Their primary function is the production of metabolic intermediates and cellular ATP through the citric acid cycle and oxidative phosphorylation pathway. For this reason, mitochondria are involved in a wide variety of cellular and developmental processes including pollen development and cytoplasmic male sterility (CMS) [1,2]. Mitochondria have their own genomes, which harbor genes for ribosomal RNAs (rRNAs), transfer RNAs (tRNAs) and subunits of the respiratory complexes. Extensive research has been performed to understand organization and function of mitochondrial genomes. To date (September 30, 2012), more than 70 plant mitochondrial genomes have been sequenced, including those of 22 seed plant species (http://www.ncbi.nlm.nih.gov/genomes/GenomesGroup.cgi?taxid=33090&opt=organelle), and of a large number of protists, algae, fungi, and animals. These studies have greatly improved our understanding of mitochondrial gene content, genome size and organization, mutation rate as well as gene shuffling events. The sequenced mitochondrial genomes exhibit significant variation in both size and actual gene content, despite the universally conserved sequence that exists between the mitochondrial genomes of diverse species [3]. The size of sequenced plant mitochondrial genomes varies more than 12-fold among angiosperms, ranging from 208 kbp in white mustard (*Brassica hitra*) [4] to over 2,700 kbp in muskmelon (*Cucumis melo*) [5], while the number of genes only varies between 50 and 69 including 30 to 37 protein-coding genes [6]. The significant variation in size of the mitochondrial genome between species is explained by expansion of the inter-genic regions, structural rearrangements and intra- or intermolecular recombination events [7]. Gene shuffling events in higher plant mitochondrial genomes have occurred due to the presence of repeated sequences [8]. In combination with sequence duplication events, this has resulted in a unique diversity of plant mitochondrial genomes [9]. Therefore, the DNA sequence of plant mitochondria has become an important tool in phylogenetics for comparison of the evolutionary relationships among species. In addition, sequencing of the mitochondrial genome has the potential to increase our understanding of the complex genetic interactions between the nuclear and the organellar genomes.

Perennial ryegrass (*Lolium perenne* L.) is a diploid (2n = 2× = 14) member of the Poaceae family and one of the most important forage and turf grass species of temperate regions worldwide [10]. Its economic importance has led to the establishment of high-density genetic maps as well as genome and transcriptome sequence resources. For example, the complete chloroplast genome sequence has recently been published [11], and assembly of the genome sequence is currently being progressed [12]. However, the complete mitochondrial genome sequence of perennial ryegrass as well as of any other forage and turf grass species was hitherto unknown.

Therefore, the main objective of this study was to sequence, assemble and annotate the perennial ryegrass mitochondrial genome. Specifically, we aimed at (i) describing the organization of the perennial ryegrass mitochondrial genome for future comparative analyses of mitochondrial genomes within *Lolium* and between closely related grass species, (ii) identifying protein-coding genes, rRNA genes, tRNA genes and open reading frames (ORFs) to understand the function the mitochondrial genome, and (iii) gaining first insights into the mitochondrial transcriptome of perennial ryegrass.

## Results

### Isolation of intact mitochondria and extraction of mtDNA

A cellular fraction containing crude mitochondria were isolated from perennial ryegrass leaf tissue by homogenization followed by differential centrifugation. Further attempts to purify the mitochondria by Percoll density gradient centrifugation failed. The crude mitochondrial fraction was characterized by measuring the activity and latency of cytochrome *c* oxidase (CCO) as a marker enzyme for the intactness of the inner mitochondrial membrane, and malate dehydrogenase (MDH), an enzyme residing in the mitochondrial matrix as well as in the cytosol and several other places in the cell [13] (Table 1). The large increase in specific CCO activity indicates that there was a 7.7-fold enrichment of mitochondria from the homogenate to the crude mitochondrial fraction as expected. The latency of CCO measures the ability of the substrate, reduced cytochrome *c*, to reach the active site of the enzyme on the outer surface of the inner membrane. The high CCO latency in both homogenate and crude mitochondria indicates that the outer membrane was mainly intact [14,15]. Only a small fraction of MDH activity co-purified with the mitochondria, but its latency increased dramatically (3.5-fold) indicating that the major part of the lost MDH had been present outside the mitochondria and that the crude mitochondria contain most of its MDH behind the permeability barrier of an intact inner membrane [16]. Thus, the crude mitochondrial fraction contained mainly intact mitochondria (90%), in which the mtDNA was protected inside intact outer and inner membranes (Table 1).

**Table 1:** Characterization of mitochondrial enrichment and intactness

**Parameters**		*Fraction	
		**Homogenate**	**Crude mitochondria**
Protein	Total (mg)	731 ± 40	42.0 ± 7
CCO activity	Total (μmol min^-1^)	13.0 ± 2.90	5.87 ± 1.41 (45%)
	Specific (nmol min^-1^ mg^-1^)	18.0 ± 2.83	139 ± 9.90 (7.7-fold)
	Latency (%)	90.0 ± 1.41	89.5 ± 0.71
MDH activity	Total (μmol min^-1^)	214 ± 61.5	26.5 ± 0.71 (12%)
	Specific (nmol min^-1^ mg^-1^)	291 ± 68.6	639 ± 91.2 (2.2-fold)
	Latency (%)	22.0 ± 1.41	77.5 ± 0.71(3.5-fold)
Yield of mtDNA	Total (μg)	-	3.47 ± 0.27

*Data are means ± SD of two separate isolations each of 30 g fresh leaves.

Subsequently, contaminating nuclear and chloroplast DNA was removed by treating the crude mitochondrial fraction with DNAse. The isolation of mtDNA from intact mitochondria from 60 g (two batches of 30 g) fresh weight leaves resulted in 3.5 μg mtDNA.

### Sequencing and assembly of the perennial ryegrass mitochondrial genome

A total of 287,367 single reads were generated with a mean length of 403 bp (approximately 116 Mbp of sequence information) from the mitochondrial genome of the perennial ryegrass genotype F1-30 using Roche 454 GS-FLX Titanium sequencing (Table 2). This resulted in a 167-fold coverage of the mitochondrial genome. The contaminating chloroplast sequence reads were removed by performing a reference assembly against the chloroplast genome (GenBank Acc. No.: NC_009950.1). The isolated mitochondrial DNA was contaminated with approximately 2% chloroplast DNA (Table 2).

**Table 2:** Summary of the perennial ryegrass mitochondrial genome sequencing and assembly

**Parameters**	**Counts**	**Average length (nucleotides per read or contig)**	**Total bases**
Total number of single reads	287,367	403	115,768,584
Total number of single reads after quality control*	281,706	403	113,583,420
Number of initial contigs	2,403	703	1,689,209
Number of mitochondrial contigs	9	80,314	722,827
Number of final contigs after closing all gaps: Genome size	1	-	678,580
Coverage	-	-	167-fold

*Removal of reads matching chloroplast DNA.

**Figure 1:**
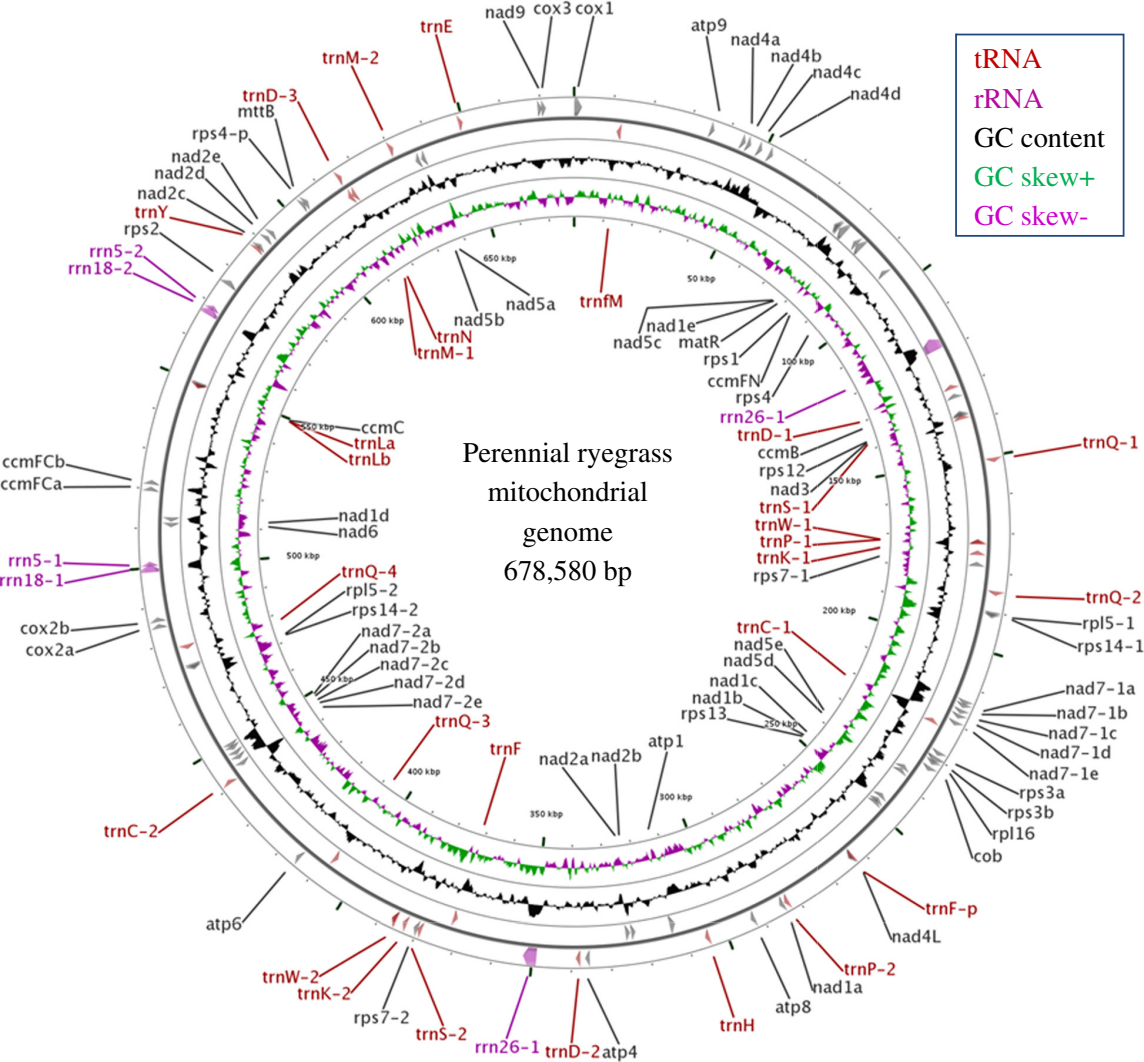
**Map of the perennial ryegrass mitochondrial genome.** Protein, tRNA and rRNA-coding genes are shown inside and outside the circles. The second outer circle represents the circular master molecule. Genes and exons are indicated by arrowheads, p indicates a pseudogene. The forward and reverse DNA strands are shown in clockwise and anticlockwise orientation, respectively. The middle black peaked circle represents the G+C content of the master molecule. The inner circle shows the size markers in kbp in clockwise orientation. The first nucleotide of the *cox*1 gene is the starting point of the circular master molecule. This figure was generated using the CGView Server [17].

The initial assembly generated 2,403 contigs totaling 1.7 Mbp with an average length of 703 nucleotides. The longest contig was 219,170 bp, and the shortest contig was 116 bp. A BLASTn search was performed against the nucleotide collection of NCBI aiming to remove contaminating contigs. Nine out of 2,403 contigs were identified as plant mitochondrial DNA sequences with a mean length of 80,314 bp (total size 722,827 bp). The remaining 2,394 contigs corresponding to 0.83% of the 116 Mbp total sequence information were contaminating sequences which was discarded. These contigs were mainly single read sequences related to other organisms (Table 2). These nine contigs of the initial assembly could not be further arranged into a single circular molecule mainly for two reasons. Firstly, there were cases of misassembled contigs, and secondly there were cases where repetitive sequences led to a breakdown of the assembly process. In order to further resolve the arrangement of the nine contigs, we made use of the sequence information available from a perennial ryegrass nuclear genome sequencing project that is ongoing in our lab, which contained assembled contigs and scaffolds originating from the mitochondrial genome. This assembly included mate-pair Illumina libraries with insert sizes of up to 9 kbp, which helped us to predict the order and orientation of a number of the nine mitochondrial contigs. This was then followed by a process of designing primers to span contigs, followed by Sanger sequencing to fill in the gaps and, ultimately, merge the contigs (Figure 1). The complete nucleotide sequence of the mitochondrial genome of perennial ryegrass has been deposited to GenBank under the accession number JX 999996.

### Features of the perennial ryegrass mitochondrial genome

The genome size was 678,580 bp with a G+C content of 44.1%. Annotation of the mitochondrial genome was performed and a total of 73 genes including protein-coding genes, rRNA genes, tRNA genes as well as 149 ORFs were identified. These regions account for 21.03% of the genome (Figure 1, Table 3, Table 4 and Additional file 1: Table S1).

**Table 3:** Main features of the assembled perennial ryegrass mitochondrial genome

Genome size (nt)	678,580
G+C content (%)	44.1
Gene number	73^a^
-Protein coding genes	39 (34)^b^ (1)^c^
-tRNA genes	28 (14)^b^ (1)^c^
-rRNA genes	6 (3)^b^
Proteins	
-Protein coding sequences (nt)	35,514
-Protein coding sequence (%)	5.23
RNAs	
-RNA gene coding sequences (nt)	13,209
-RNA gene coding sequences (%)	1.95
Introns	
-Cis-spliced group II introns	22
-Trans-spliced group II introns	4
-Introns sequences of these genes (nt)	26,631
-Introns of these genes sequences (%)	3.92
ORFs	
-No. of ORFs	149
-ORFs (>300 bp) sequences (nt)	67,410
-ORFs sequences (%)	9.93
Total (proteins, RNAs and ORFs) sequences	142,764
Total (proteins, RNAs and ORFs) sequences (%)	21.03
Repeats	
-Large repeat sequences (nt)	116,177
-Large repeat sequences (%)	17.12
Transposable element-related sequences (%)	3.76

^a^Total numbers including duplications and pseudogenes, ^b^In parentheses, number of unique genes, ^c^ In parentheses, number of pseudogenes.

**Table 4:** Genes identified in the perennial ryegrass mitochondrial genome

**Name of the gene**^**a**^	**Size**^**b **^**(bp)**	**Position in the genome**		**Strand**^**c**^	**No. of amino acids**	
		**From**	**To**			
A. Protein and rRNA-coding genes						
1. Complex I genes						
*nad*1	**978**	-	-	x	**325**	
*nad*1a	387	284,188	284,574	+	129	
*nad*1b	81	247,595	247,675	-	27	
*nad*1c	192	245,995	246,186	-	64	
*nad*1d	60	512,368	512,427	-	20	
*nad*1e	258	76,540	76,797	-	85	
*nad2*	**1,467**	-	-	x	**488**	
*nad*2a	153	324,853	325,005	-	51	
*nad*2b	396	323,224	323,619	-	132	
*nad*2c	159	588,650	588,809	+	53	
*nad*2d	573	591,214	591,786	+	191	
*nad*2e	186	593,194	593,381	+	61	
*nad*3	357	137,432	137,788	-	118	
*nad*4	**1,488**	-	-	+	**495**	
*nad*4a	461	43,953	44,413	+	153	
*nad*4b	515	45,442	45,956	+	172	
*nad*4c	423	48,576	48,998	+	141	
*nad*4d	89	51,790	51,878	+	29	
*nad*4L	315	263,099	263,413	+	104	
*nad5*	**2,013**	-	-	-	**670**	
*nad*5a	228	637,049	637,277	-	76	
*nad*5b	1,218	634,967	636,186	-	406	
*nad*5c	21	76,157	76,177	-	7	
*nad*5d	396	237,378	237,773	-	132	
*nad*5e	150	236,297	236,446	-	49	
*nad*6	945	510,437	511,381	-	314	
*nad7-1*	**1,185**	-	-	+	**394**	
*nad*7-1a	143	213,945	214,087	+	47	
*nad*7-1b	69	214,890	214,958	+	23	
*nad*7-1c	467	216,269	216,735	+	155	
*nad*7-1d	244	217,735	217,978	+	81	
*nad*7-1e	262	219,684	219,945	+	86	
*nad*7-2	**1,185**	-	-	-	**394**	
*nad*7-2a	143	449,072	449,214	-	47	
*nad*7-2b	69	448,201	448,269	-	23	
*nad*7-2c	467	446,424	446,890	-	155	
*nad*7-2d	244	445,181	445,424	-	81	
*nad*7-2e	262	443,214	443,475	-	86	
*nad*9	573	669,561	670,133	+	190	
2. Complex III gene						
*cob*	1,194	232,540	233,733	+	397	
3. Complex IV genes						
*cox1*	1,989	1	1,989	+	662	
*cox*2	**783**	-	-	+	**260**	
*cox*2a	388	484,835	485,222	+	130	
*cox*2b	395	486,506	486,897	+	130	
*cox*3	843	670,403	671,245	+	280	
4. Complex V genes						
*atp*1	1,530	312,019	313,548	-	509	
*atp*4	588	335,648	336,235	+	195	
*atp*6	714	415,086	415,799	+	237	
*atp*8	468	292,308	292,775	+	155	
*atp*9	225	35,605	35,829	+	74	
5. Cytochrome *c*						
biogenesis genes						
*ccm*B	651	132,035	132,685	-	216	
*ccm*C	723	549,113	549,835	-	240	
*ccm*FN	1,770	83,571	85,340	-	589	
*ccm*FC	**1,434**	-	-	+	**477**	
*ccm*FCa	755	519,648	520,402	+	252	
*ccm*FCb	679	521,399	522,077	+	225	
6. Ribosomal proteins						
genes						
*rps*1	636	82,801	83,436	-	211	
*rps*2	1,464	575,833	577,296	+	487	
*rps*3	**1,650**	-	-	+	**549**	
*rps*3a	72	228,586	228,657	+	24	
*rps*3b	1,578	229,590	231,167	+	525	
*rps*4	1,035	93,583	94,617	-	344	
*rps*4-p	1,032	603,531	604,562	+	343	
*rps*7-1	447	177,808	178,254	-	148	
*rps*7-2	447	380,279	380,725	+	148	
*rps*12	378	137,010	137,387	-	125	
*rps*13	351	248,209	248,559	-	116	
*rps*14-1	159	190,863	191,021	+	52	
*rps*14-2	159	472,138	472,296	-	52	
*rpl*5-1	552	190,178	190,729	+	183	
*rpl*5-2	552	472,430	472,981	-	183	
*rpl16*	558	231,058	231,615	+	185	
7. Other protein coding genes						
*mat*R	1,977	77,314	79,290	-	658	
*mtt*B	816	604,582	605,397	+	271	
8. rRNA genes						
*rrn*5-1	122	500,941	501,062	+	-	
*rrn*5-2	122	568,955	569,076	+	-	
*rrn*18-1	1,966	498,862	500,827	+	-	
*rrn*18-2	1,966	566,875	568,841	+	-	
*rrn*26-1	3,461	115,757	119,217	-	-	
*rrn*26-2	3,457	348,904	352,360	+	-	
**Name of the gene**^**a**^	**Size**^**b **^**(bp)**	**Position in the genome**		**Strand**^**b**^	**tRNA type**	**Anti-codon**
		**From**	**To**			
B. tRNA-coding genes						
*trn*N	72	616,184	616,255	-	Asn	GTT
*trn*D-1	74	129,749	129,822	-	Asp	GTC
*trn*D-2	74	338,298	338,371	+	Asp	GTC
*trn*D-3	74	615,471	615,544	+	Asp	GTC
*trn*C-1	71	221,721	221,791	-	Cys	GCA
*trn*C-2	71	441,367	441,437	+	Cys	GCA
*trn*Q-1	72	151,087	151,158	+	Gln	TTG
*trn*Q-2	72	185,132	185,203	+	Gln	TTG
*trn*Q-3	72	407,374	407,445	-	Gln	TTG
*trn*Q-4	72	477,956	478,027	-	Gln	TTG
*trn*E	73	649,329	649,401	+	Glu	TTC
*trn*H	74	304,917	304,990	+	His	GTG
*trn*L	**71**	-	-	-	Leu	CAA
*trn*La	37	549,042	549,076	-		
*trn*Lb	34	548,992	549,029	-		
*trn*K-1	73	174,964	175,036	-	Lys	TTT
*trn*K-2	73	383,497	383,569	+	Lys	TTT
*trn*fM	74	11,905	11,978	-	Met	CAT
*trn*M-1	74	615,054	615,127	-	Met	CAT
*trn*M-2	73	630,497	630,569	+	Met	CAT
*trn*F	73	371,486	371,558	-	Phe	GAA
*trn*P-1	74	172,177	172,250	-	Pro	TGG
*trn*P-2	75	282,933	283,007	+	Pro	TGG
*trn*P-3	74	386,282	386,355	+	Pro	TGG
*trn*S-1	88	138,301	138,388	-	Ser	GCT
*trn*S-2	86	379,430	379,515	+	Ser	TGA
*trn*W-1	74	171,961	172,034	-	Trp	CCA
*trn*W-2	74	386,498	386,571	+	Trp	CCA
*trn*Y	83	587,933	588,015	+	Tyr	GTA
*trn*F-p	73	262,935	263,007	+	Phe	GAA

A total of 39 protein-coding genes and 6 rRNA-coding genes (**A**) and 28 tRNA-coding genes (**B**) were identified. ^a^p, pseudogene; ^b^Boldface, sum of all exons; lower-case letters, exons of protein-coding genes; hyphenated, duplicate genes; ^c^Plus and minus, encoded by the forward and reverse DNA strand, respectively; x, trans-spliced.

### Protein-coding genes

The perennial ryegrass mitochondrial genome contains 39 genes encoding 34 different proteins including one pseudogene, *rps*4-p (Table 4). The genes encode 19 proteins of the electron transport chain. They include nine subunits of complex I: NADH dehydrogenase subunits 1, 2, 3, 4, 4L, 5, 6, 7 and 9 (*nad*1, 2, 3, 4, 4L, 5, 6, 7 and 9) of which *nad*7 has two copies; one subunit of complex III: apocytochrome b (*cob*); three subunits of complex IV: cytochrome *c* oxidase subunits 1, 2 and 3 (*cox*1, 2 and 3); five subunits of complex V: ATP synthase F1 subunits 1, 4, 6, 8 and 9 (*atp*1, 4, 6, 8 and 9). No genes were found to encode subunits of complex II: succinate dehydrogenase subunits 3 and 4 (*sdh*3 and *sdh*4). In addition, four genes encode proteins involved in cytochrome *c* biogenesis: subunits B, C and F (*ccm*B, C, FN and FC). Two genes, *mat*R and *mtt*B, encode maturase and transport membrane proteins, respectively. The thirteen genes *rps*1, *rps*2, *rps*3, *rps*4, *rps*7-1, *rps*7-2, *rps*12, *rps*13, *rps*14-1 and *rps*14-2, and *rpl*5-1, *rpl*5-2 and *rpl*16 encode ribosomal proteins. In total, the protein-coding regions cover 5.23% of the mitochondrial genome (Figure 1, Table 3 and Table 4).

### RNA-coding genes

The perennial ryegrass mitochondrial genome contains a total of 34 RNA-coding genes: three rRNA genes (present in two copies each) for the ribosomal subunits 18 S, 26 S and 5 S, and 28 tRNA genes including one pseudogene, tRNA-Phe^GAA^ (Table 4). The anticodon of 28 tRNA genes match the codons of a total of 14 amino acids. The RNA-coding genes represent 1.95% of the mitochondrial genome (Figure 1, Table 3). The length of the rRNA genes range from 122 to 3,461 nucleotides, and the tRNA genes range from 71 to 88 nucleotides (Table 4). No tRNA genes were found for the amino acids Alanine (Ala), Arginine (Arg), Glysine (Gly), Isoleucine (Ile) Threonine (Thr), and Valine (Val) in the perennial ryegrass mitochondrial genome (Table 5).

**Table 5:** Differences in the tRNA gene content in sequenced mitochondrial genomes of grasses

**tRNA genes**	**Plant species**								
	***Lolium perenne***	***Ferrocalamus rimosivaginus***	***Bambusa oldhamii***	***Triticum aestivum***	***Oryza sativa***	***Oryza rufipogon***	***Sorghum bicolor***	***Zea mays***	***Zea perennis***
*trn*A	-	-	-	-	-	-	-	-	-
*trn*G	-	-	-	-	-	-	-	-	-
*trn*P	+	+	+	+	+	+	+	+	+
*trn*T	-	-	-	-	-	-	-	-	-
*trn*V	-	-	-	-	-	-	-	-	-
*trn*S	+	+	+	+	+	+	+	+	+
*trn*R	-	-	-	-	-	-	-	-	-
*trn*L	+	-	-	-	-	-	-	-	-
*trn*F	+	+	+	+	+	+	+	+	+
*trn*N	+	+	+	+	+	+	+	+	+
*trn*K	+	+	+	+	+	+	+	+	+
*trn*D	+	+	+	+	+	+	+	+	+
*trn*E	+	+	+	+	+	+	+	+	+
*trn*H	+	+	+	-	+	+	+	+	+
*trn*Q	+	+	+	+	+	+	+	+	+
*trn*I	-	+	+	+	+	+	+	+	+
*trn*M	+	+	+	+	+	+	+	+	+
*trn*W	+	+	+	+	+	+	+	-	-
*trn*C	+	+	+	+	+	+	+	+	+
*trn*Y	+	+	+	+	+	+	+	+	+
*trn*fM	+	+	+	+	+	+	+	+	+

The accession numbers of the mitochondrial genomes are: *Ferrocalamus rimosivaginus* (JQ235166 to JQ235179), *Bambusa oldhamii* (EU365401), *Triticum aestivum* (AP008982), *Oryza sativa* (BA000029), *O. rufipogon* (NC_013816), *Sorghum bicolor* (NC_008360), *Zea mays* (AY506529) and *Z. perennis* (NC_008331). +, present; -, absent.

### Introns

Among the 39 protein-coding genes, only nine (*nad*1, *nad*2, *nad*4, *nad*5, *nad*7-1, *nad*7-2, *cox*2, *ccm*FC and *rps*3) contain introns. All the introns in sequenced mitochondrial genomes are classified as group II introns [18]. A total of 26 group II introns were found within the nine protein-coding genes including four trans-spliced introns that are part of *nad*1 and *nad*2 (Table 4). In total, twenty-two cis-spliced introns are present in *nad*1, *nad*2, *nad*4, *nad*5, *nad*7, *cox*2, *ccm*FC and *rps*3. Among 28 tRNA genes, one tRNA gene *trn*L^CAA^ contains an intron.

### Open reading frames (ORFs)

An ORF may be defined as an in-frame DNA sequence of 300 bp or longer that is bordered by a start and stop codon, with no match to a coding sequence in the public databases [19]. In the perennial ryegrass mitochondrial genome, we found 149 ORFs with a minimum and maximum length of 303 and 2,571 bp, respectively, and with a mean length of 452 bp covering 9.93% of the genome (Table 3, Additional file 1: Table S1).

### Repetitive regions and their gene content

**Figure 2:**
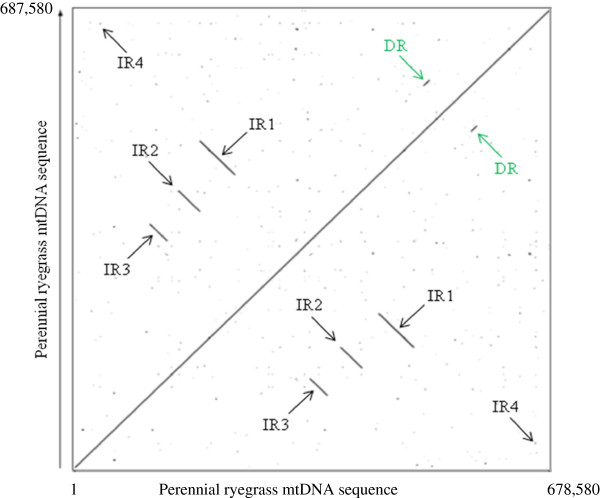
**Dot plot of the perennial ryegrass mitochondrial genome.** Five repeats including four large inverted repeat pairs, IR1-IR4 and one direct repeat, DR (green) are marked by arrows. The inverted and direct repeat coordinates in the master molecule are: IR1, (178,334-228,600 bp; 484,825-434,554 bp); IR2, (147,726-178,558 bp; 410,808-379,975 bp); IR3, (106,923-131,907 bp; 361,199-336,212 bp); IR4 (94,022-95,555 bp; 604,126-602,594 bp) and DR (498,590-507,147 bp; 566,604-575,159 bp). This figure was generated using the PipMaker software [20].

A variety of repetitive DNA sequences were found in the perennial ryegrass mitochondrial genome. There are four pairs of large inverted repeat (IR) sequences, with repeat lengths of 50,267, 30,833, 24,985 and 1,534 bp, as well as one large directly repeated (DR) sequence of 8,558 bp (Figure 2), with 99% sequence identity. Overall, these five large repeats account for 17.12% of the mitochondrial genome. The genes, *nad*7, *rps*7, *rps*14, *rpl*5, *rrn*5, *rrn*18, *rrn*26, *trn*D, *trn*C, *trn*Q, *trn*K, *trn*M, *trn*P, *trn*S and *trn*W, were found as multiple copies located in the large inverted repeat sequences in the perennial ryegrass mitochondrial genome (Figure 2, Table 4).

A total of 96 pairs of short inverted repeat (SIR) sequences were identified covering 4,886 bp (0.72%) of the mitochondrial genome. Percent matches between SIRs were higher than 68%, with 83 pairs of SIRs showing values higher than 80%, while thirteen pairs of SIRs had a sequence identity of 100%. The average SIR length was 52 bp, and the longest SIR identified was 333 bp (Additional file 2: Table S2).

The mitochondrial genome of perennial ryegrass contained 29 tandem repeats, which covered 1,647 bp corresponding to 0.24% of the total sequence. The average period size and copy number were 26.07 and 2.17, respectively. The most common type of tandem repeat had period sizes of 42, 14 and 12, which totaled 42% of all tandem repeats found in the mitochondrial genome (Additional file 3: Table S3).

### Simple sequence repeat sequences

We found 250 SSRs in the perennial ryegrass mitochondrial genome including 23, 196, 26 and 5 with mono-, tri-, tetra- and pentanucleotide repeats, respectively. SSRs with dinucleotide repeats were not found. The length of the mononucleotide, trinucleotide and pentanucleotide repeats range from 10–13, 9–12 and 15–20 bp, respectively. All the tetranucleotide repeats are 12 bp long (Additional file 4: Table S4). Of 196 trinucleotide repeats, only 14 (7.14%) were present in the coding regions in the perennial ryegrass mitochondrial genome.

### Transposable element-related sequences

The presence of transposable elements (TEs) was also investigated using two different TE databases; Poaceae and Triticeae as queries from the Genetic Information Research Institute (http://www.girinst.org/censor/index.php). In total, 22,545 bp (3.32%) and 3,008 bp (0.44%) of the total genome sequence showed homology with TEs in Poaceae and Triticeae, respectively. The TEs were mainly long terminal retrotransposon (LTR) elements (Table 6). The circular master molecule coordinates of the TEs are presented in Additional file 5: Table S5.

**Table 6:** Transposable elements found in the perennial ryegrass mitochondrial genome

**Repeat class**	^**a**^**Poacece**		^**a**^**Triticeae**	
	**Fragments**	**Length**	**Fragments**	**Length**
Transposable element				
1.DNA transposon	**12**	**1,125**	**3**	**324**
-EnSpm	3	220	3	324
-Helitron	4	329	-	-
-MuDR	2	118	-	-
-hAT	1	253	-	-
2.LTR retrotransposon	**56**	**16,758**	**17**	**2,566**
-Copia	20	4,939	3	153
-Gypsy	36	11,819	14	2,413
3.Non-LTR retrotransposon	**27**	**4,662**	**2**	**118**
-L1	27	4,662	2	118
Total	**95**	**22,545**	**22**	**3,008**

^a^Reference DNA; Bold face, sum of fragments and lengths.

### Transcriptome analyses

We performed an expression analysis of the 39 mitochondrial protein-coding genes (Table 7) using in-house RNA-seq data from the reproductive tissue of perennial ryegrass inflorescence (unpublished). The results are presented both as total number of reads matching the genes or as the number of reads corrected for the gene length (normalized expression). The most abundantly expressed genes in terms of total numbers were *cob*, *cox*1 and *atp*1, which all had more than 10,000 matching reads. However, when comparing normalized expression, the most highly expressed genes were *nad*9, *cob*, *cox*3, *atp*9 and *rps*12 with more than 10,000 reads per kbp gene length. Eleven genes had low expression (<1,000 matching reads per kbp gene length), namely *ccm*B, *rps*4, *rps*4-p, *rps*7-1, *rps*7-2, *rps*14-1, *rps*14-2, *rpl*5-1, *rpl*5-2, *mat*R and *mtt*B. Of these, the genes *rps*14-1, *rps*14-2, *rpl*5-1, and *rpl*5-2 had fewer than 100 matching reads per kbp gene length. The genes encoding subunits of the respiratory complexes (*nad*1 to *nad*9, *cob*, *cox*1 to *cox*3 and *atp*1 to *atp*9) all showed high expression both in absolute numbers and after normalization. In contrast, the genes encoding ribosomal proteins varied enormously in their expression levels (Table 7).

**Table 7:** Expression profile of the protein-coding genes in the perennial ryegrass mitochondrial genome

**Genes**	**RNA-seq read counts**	**Coding sequences (kbp)**	**Relative expression**
*nad*1	2,392	0.975	2,453
*nad*2	4,573	1.464	3,124
*nad*3	2,816	0.354	7,955
*nad*4	5,967	1.485	4,018
*nad*4L	1,391	0.312	4,458
*nad*5	8,251	2.010	4,105
*nad*6	2,426	0.942	2,575
*nad*7-1	2,766	1.182	2,340
*nad*7-2	2,766	1.182	2,340
*nad*9	6,974	0.570	12,236
*cob*	12,226	1.191	10,265
*cox*1	17,574	1.986	8,849
*cox*2	6,083	0.780	7,799
*cox*3	9,713	0.840	11,564
*atp*1	15,214	1.527	9,963
*atp*4	2,388	0.585	4,082
*atp*6	4,633	0.711	6,516
*atp*8	3,454	0.465	7,429
*atp*9	5,361	0.222	24,150
*ccm*B	235	0.648	363
*ccm*C	1,327	0.720	1,843
*ccm*FC	1,702	1.431	1,190
*ccm*FN	3,739	1.767	2,116
*rps*1	1,645	0.633	2,599
*rps*2	1,635	1.461	1,119
*rps*3	9,172	1.647	5,569
*rps*4	503	1.032	487
*rps*4-p	424	1.029	412
*rps*7-1	205	0.444	461
*rps*7-2	205	0.444	461
*rps*12	5,072	0.375	13,526
*rps*13	388	0.348	1,115
*rps*14-1	7	0.156	42
*rps*14-2	7	0.156	42
*rpl*5-1	52	0.549	96
*rpl*5-2	52	0.549	96
*rpl*16	4,963	0.555	8,942
*mat*R	416	1.974	211
*mtt*B	698	0.813	859
Total	149,414	35.514	

Gene name, RNA-seq read counts, protein-coding gene length in kbp, and the relative expression.

## Discussion

### Intactness of mitochondria

To obtain mtDNA uncontaminated by nuclear or chloroplast DNA, crude but intact mitochondria were isolated. Because the mtDNA is located behind two permeability barriers in intact mitochondria – an intact inner membrane as monitored by MDH latency [16] and an intact outer membrane as monitored by CCO latency [14,15] – treatment with DNAse removed contaminating chloroplastic and nuclear DNA without degrading the mtDNA. The fact that only 2% of the sequenced contigs were chloroplastic (Table 2) shows that this strategy was successful.

### Difficulties of *de novo* assembly of a plant mitochondrial genome

Although the average length of the gaps between the contigs was only 122 bp, it was not straightforward to close the gaps through PCR amplification of the missing DNA segments. This was mainly due to misassembled, duplicated and repetitive DNA sequences in the nine *de novo* assembled contigs. Few reports have been published on mitochondrial genome sequencing using next-generation sequencing due to assembly difficulties of short reads even when a reference genome exists [6].

Many next-generation sequencing platforms produce paired-end or mate-pair reads, which collectively can be referred to as read-pairs. Because the approximate physical distance of the read pairs are known, the paired nature of these reads constitutes a powerful source of information, significantly facilitating genome assembly, because they can span repetitive regions, and therefore can be used to join contigs.

It is currently not possible to isolate nuclear DNA from plants without having the nuclear DNA contaminated with organellar DNA (mitochondrial and chloroplast). Thus, organellar genomes are also to a certain degree being sequenced as part of a nuclear genome sequencing project, and prior to filtering, the perennial ryegrass genome draft therefore also contains mitochondrial contigs and scaffolds. The mitochondria-related scaffold information was used to re-assemble, order and orientate the mitochondrial contigs in our mitochondrial genome assembly, and for primer design to facilitate PCR amplification across gaps.

### Features of the perennial ryegrass mitochondrial genome

The final assembly of the perennial ryegrass mitochondrial genome resulted in a single circular molecule of 678,580 bp with an average G+C content of 44.1% (Figure 1, Table 3). The G+C content is very similar to that of other sequenced plant mitochondrial genomes such as rice (*Oryza sativa* L.), 43.8%; bamboo (*Ferrocalamus rimosivaginus* L*.*), 44.1%; sugar beet (*Beta vulgaris* L.), 43.9%; melon (*Cucumis melo* L.), 44.5%; Arabidopsis (*Arabidopsis thaliana* L.) 44.8% and rapeseed (*Brassica napus* L.), 45.2% [5,7,21-25]. The perennial ryegrass mitochondrial genome contains 73 genes including genes encoding known proteins and RNAs. Among the identified genes, 36 genes (30 encode for proteins, and 6 for tRNAs) are single-copy genes. The remaining 35 genes are multi-copy genes of *nad*7, *rps*7, *rps*14, *rpl*5, *rrn*5, *rrn*18, *rrn*26, *trn*D, *trn*C, *trn*Q, *trn*K, *trn*M, *trn*P, *trn*S, *trn*W and two pseudogenes, *rps*4-p and *trn*F-p (Table 4, Table 8). The 73 genes gave a density of one coding region per 9.30 kbp, which is less compact than bamboo, rice and *Arabidopsis* (one coding region per 7.73, 7.91 and 8.0 kbp, respectively) [21,22,25]. Gene distribution between two DNA strands depends on the different genomic configuration [26], but generally shows no extreme strand bias [25]. In the perennial ryegrass mitochondrial genome, two protein-coding genes, *nad*1 and *nad*2 are trans-spliced, 21 genes are encoded on the forward strand, and 16 on the reverse strand. All the rRNA genes are located on the forward strand except the *rrn*26-1 gene, while 15 tRNA genes are found on the forward strand and 13 on the reverse strand (Figure 1, Table 4).

**Table 8:** Copy number of mitochondrial genes that differ in perennial ryegrass, bamboo, wheat, rice and maize

**Genes**	**Perennial ryegrass**	**Bamboo**^**a**^	**Wheat**^**a**^	**Rice**^**a**^	**Maize**^**a**^
*atp*1	1	1	1	2	2
*atp*4	1	1	1	2	1
*atp*6	1	1	2	1	1
*atp*8	1	1	2	1	1
*cox*3	1	1	1	2	1
*nad*1a	1	1	1	2	2
*nad*2c	1	1	1	2	1
*nad*2d,e	1	1	1	2	2
*nad*4d	1	1	1	3	1
*nad*5a,b	1	1	1	2	1
*nad*7	2	1	1	1	1
*nad*9	1	1	1	2	1
*rpl*2	0	0	0	3	0
*rpl*5	2	1	1	2	0
*rps*2	1	1	1	1	2
*rps*3a	1	1	1	1	2
*rps*7	2	1	1	1	1
*rps*14	2	0	0	0	0
*rps*19	0	0	0	1	0
*rrn*5	2	1	3	2	1
*rrn*18	2	1	3	2	1
*rrn*26	2	1	2	2	1
*trn*C	2	0	0	0	0
*trn*D	3	1	2	1	2
*trn*E	1	1	1	1	2
*trn*fM	1	1	3	1	1
*trn*I	0	1	1	1	2
*trn*K	2	1	3	1	1
*trn*M	2	0	1	1	0
*trn*N	1	0	0	1	1
*trn*P	3	1	2	1	2
*trn*Q	4	1	3	1	1
*trn*W	2	0	0	0	0

Gene fragments, pseudogenes and chloroplast-derived genes are excluded. Genes with the same number of copies in all five species are not included. ^a^From bamboo (Acc. No. JQ235166 to JQ235179) [22], rice (Acc. No. BA000029) [21], maize (Acc. No. AY506529) [19], and wheat (Acc. No. AP008982) [27]. Lower case letter indicates exons of this gene.

The coding and intron sequences occupy 7.2% (48,723 bp) and 3.9% (26,631 bp) of the genome, respectively, including 39 protein, 28 tRNA and 6 rRNA genes (Table 3). In the maize (*Zea mays* L.) NB mitochondrial genome coding sequences account for 6.22% of the total genome [19]. Generally, the functional mitochondrial rRNA and tRNA genes of the sequenced angiosperm mitochondrial genomes lack introns [19]. We found that one tRNA gene, *trn*L^CAA^ contains an intron in the perennial ryegrass mitochondrial genome. Similar results have been found in the date palm (*Phoenix dactylifera* L.) mitochondrial genome where three tRNA genes, *trn*K^TTT^, *trn*N^ATT^ and *trn*Sup^CTA^, also contained an intron [28]. Further work is needed to determine if the perennial ryegrass intron containing tRNA gene, *trn*L^CAA^ is functional.

The variation in the number of mitochondrial genes between species is mainly due to differences in gene content for the subunits of complex II, and especially ribosomal proteins and tRNAs [18]. Multiple copies of rRNA genes were found in perennial ryegrass (Table 8), and also in the mitochondrial genomes of sugar beet and wheat (*Triticum aestivum* L.) [23,27]. All the known respiratory genes, except for the complex II genes *sdh*3 and *sdh*4, are present in the perennial ryegrass mitochondrial genome (Table 4). Both *sdh*3 and *sdh*4 genes are functional in tobacco and melon [5,29] but absent in all other species, as reviewed by Ma et al. [22]. *Sdh*4 has been identified as a pseudogene in *Arabidopsis*, rapeseed and sugar beet [7,23,25]. Although the perennial ryegrass mitochondrial genome contains some multi-copy genes, it lacks the *rpl*2, *rps*10, *rps*11 and *rps*19 genes (Table 4, Table 8). *Rpl*2 is missing in sorghum (*Sorghum bicolor* L.), *Tripsacum*, maize and sugar beet [22]; it is functional in *Arabidopsis*, rice, rapeseed, tobacco and melon [5,7,21,25,29]; and it is a pseudogene in wheat and bamboo [22,27]. *Rps*10 is only present in tobacco and melon [5,29]. The *rps*11 gene is present in liverwort (*Marchantia polymorpha*) but has not been found in eudicotyledonous and monocotyledonous species except for the rice mitochondrial genome which retains *rps*11 as a pseudogene [21,30]. The *rps*19 gene is functional in rice, bamboo, and tobacco, and has been identified as a pseudogene in wheat and *Arabidopsis*. In the perennial ryegrass mitochondrial genome, we found multiple copies of the *rps*14 gene, which has only been identified as a functional gene in rapeseed [7]. The comparison of all 14 ribosomal protein and both complex II (*sdh*) genes in 280 diverse angiosperms has demonstrated frequent loss of some of these 16 mitochondrial genes during angiosperm evolution [31]. It seems that genes encoding ribosomal proteins and complex II proteins are species-specific. In order to understand the gene loss and gain event in the angiosperm mitochondrial genome, it might be interesting to know the compensation pathway of the genes which are missing in the mitochondrial genome. The compensation pathway might be the first reason that genes which are no longer necessary to function in the cell can disappear entirely from the cell. The second reason is gene substitution or gene replacement, where the function of the missing mitochondrial gene is still needed and is directly replaced by a preexisting nuclear gene whose product can play the same role in the mitochondrion [32].

The perennial ryegrass mitochondrial genome has 3 rRNA genes, *rrn*18, *rrn*5 and *rrn*26, encoding the small subunit and large subunit rRNAs, which are present in all characterized mitochondrial genomes. The *rrn*5 gene is very small (122 nucleotides). In contrast to the rRNA genes in the mitochondrial genome, the mitochondrial 5S rRNA gene is absent in the mitochondrial genome of some fungi, animals and protists [3], and present only in the lands plants, a subset of algae [33] and in the protozoan [34].

Plant mitochondrial genes are translated using the universal genetic code and require tRNAs for all 20 amino acids, and the composition of the tRNA genes, encoded by the plant mitochondrial genomes, are to a high degree unique in angiosperms. In the perennial ryegrass mitochondrial genome, 27 functional tRNA genes are found for 14 amino acids. One pseudogene, *trn*F-p remains non-functional in the genome. Post-transcriptional modification within the anticodon sequence might be necessary to generate a functional *trn*F-p gene. Thus, functional tRNA genes for six essential amino acids, Ala, Arg, Gly, Ile, Thr and Val, are missing from the perennial ryegrass mitochondrial genome (Table 5), although tRNAs for 20 amino acids are required for protein synthesis in mitochondria. The missing six are presumably encoded by the nuclear genome and imported from the cytosol into the mitochondria [35,36]. The tRNA gene content of the perennial ryegrass mitochondrial genome was compared with eight other grass mitochondrial genomes, and differences were observed with respect to presence or absence of tRNAs for Ala, Arg, Gly, Ile, leu, Thr, Trp and Val among these plant species. Plastid-derived tRNAs were not considered in the comparison (Table 5). In the perennial ryegrass mitochondrial genome, twenty-four tRNAs display a classical clover leaf structure, whereas each of the two tRNA-Ser (tRNA-Ser^TGA^ and tRNA-Ser^GCT^) fold into an unusual four-loop secondary structure. One of the tRNAs (tRNA-Tyr ^GTA^) has a two stem clover leaf structure. In the maize NB mitochondrial genome, tRNA-Ser^GCU^ and tRNA-Ser^UGA^ have a five loop secondary structure [19].

In addition to protein and RNA-coding genes, we identified 149 ORFs larger than 300 bp in the perennial ryegrass mitochondrial genome (Additional file 1: Table S1). Only ORFs found outside the genic regions of the mitochondrial genome were included in the analysis. The number of ORFs larger than 300 bp identified in the perennial ryegrass mitochondrial genome are comparable to previously reported for other species such as maize (121), sugar beet (93), *Arabidopsis* (85), rice (461), wheat (179) and tobacco (110) [19,24,25,27,29,37].

### Gene content in the repetitive regions

The mitochondrial genome of perennial ryegrass contains multiple copies of the genes *nad*7, *rps*7, *rps*14, *rpl*5, *rrn*5, *rrn*18, *rrn*26, *trn*D, *trn*C, *trn*Q, *trn*K, *trn*M, *trn*P, *trn*S and *trn*W (Table 4, Table 8). All of the multi-copy protein genes are located in the inverted repeat regions, and multi-copy RNA genes are located in both repeat and inverted repeat regions (Figure 2). As for *trn*P, two copies were identical, whereas the third copy differed by a single nucleotide. The *trn*M-1 also differed from *trn*M-2 by a single nucleotide. Similarly, as for *trn*Q in the wheat mitochondrial genome, two copies are identical, whereas the third copy differed by a single nucleotide [27]. A comparison of multi-copy mitochondrial genes among grass genomes such as ryegrass, bamboo, wheat, rice and maize (Table 8), suggests that gene duplication is a species-specific phenomenon [27]. The large repeated sequences covers 17.35% of the maize NB mitochondrial genome [19], while such sequences covered 17.12% in perennial ryegrass.

### Splicing

Splicing is often part of post-transcriptional modification of messenger RNAs (mRNAs). It involves the excision of non-coding intron sequences from a precursor RNA and subsequently ligation of the flanking exon sequences to produce a mature transcript. Two types of splicing were found in the perennial ryegrass mitochondrial genome: cis-splicing, the intramolecular ligation of exon sequences on the same precursor RNA, and trans-splicing involving the intermolecular ligation of exon sequences from different primary transcripts [38]. Trans-splicing is characteristic for angiosperm mitochondrial introns, particularly for genes encoding complex I subunits [18]. Of 26 introns of the protein-coding genes, only four were trans-spliced (*nad*1 and *nad*2), confirming that trans-slicing is less common than cis-spicing [38]. In the perennial ryegrass mitochondrial genome, two genes *nad*1 and *nad*2, encoding proteins of NADH dehydrogenase subunits 1 and 2, were each split into 5 exons. In *nad*1, exon *nad*1a was located far from the other four exons on the other stand. In case of the *nad*2 gene, exons *nad*2a and *nad*2b were found approximately 265 kb from the other three exons on the other strand.

### Genome diversity

The mitochondrial genomes of flowering plants are more complex than those of animal and fungi [39,40]. They extensively vary in size (ranging from 208 kbp in white mustard [4] to over 2,700 kbp in muskmelon [5]), gene content, genome rearrangement patterns and presence of repetitive sequences. Multiple copies of a few of the conserved full-length genes or exons are found in the mitochondrial genome (Table 8), which has also undergone size expansion when compared between plant species. In the perennial ryegrass mitochondrial genome, only 142,764 bp (21.03%) of the total DNA sequences encode proteins, RNAs and ORFs (Table 3), while the vast majority of the genome sequence has unknown function.

**Figure 3:**
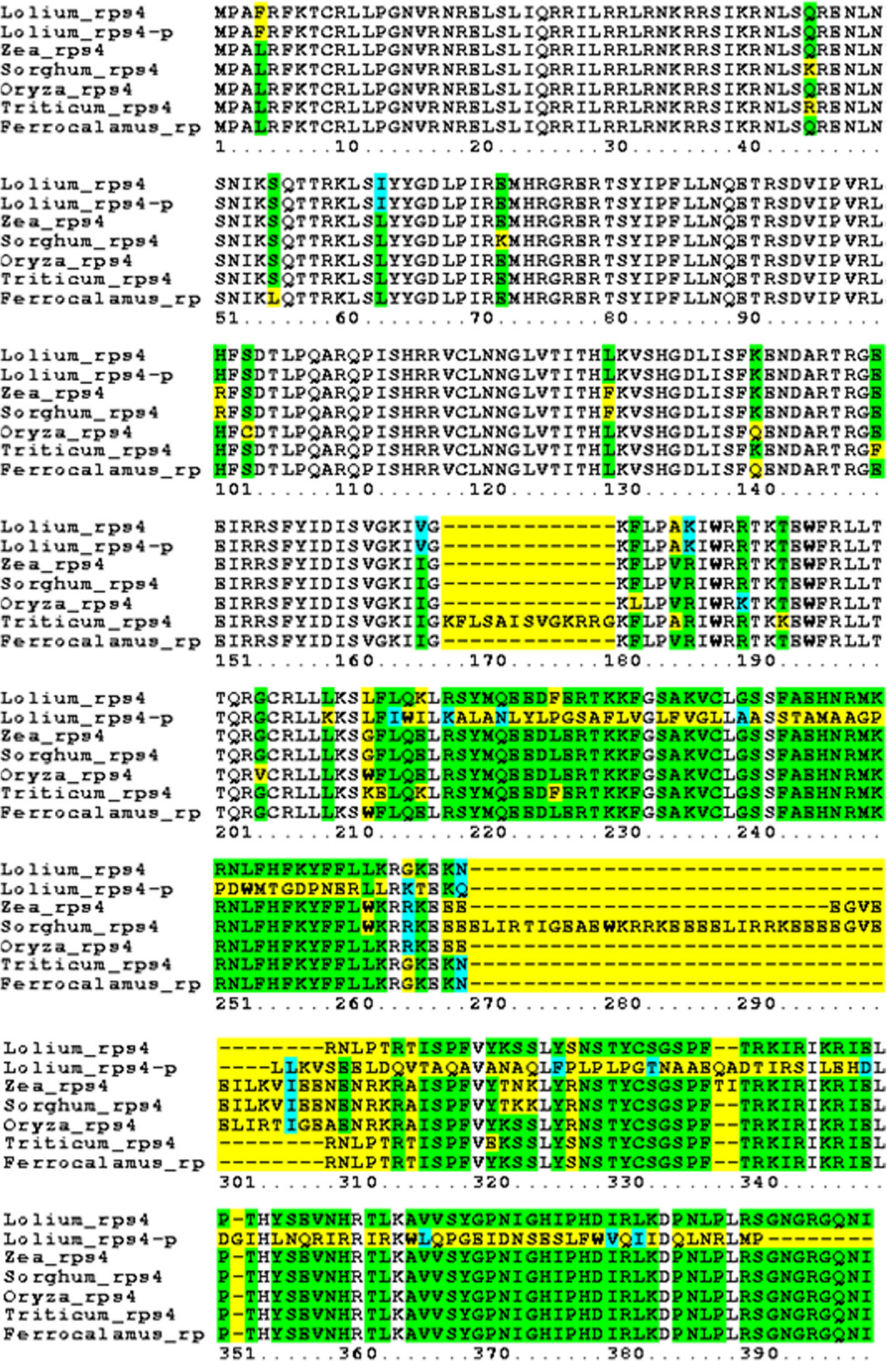
**Amino acid sequence alignment of the perennial ryegrass *rps*4 and *rps*4-p genes with the *rps*4 gene of maize, sorghum, rice, wheat and bamboo.** Amino acid sequence of the *rps*4 gene of maize (Acc. No. YP_588274.1), sorghum (Acc. No. YP_762343.1), rice (Acc. No. YP_514660.1), wheat (Acc. No. ADE08097.1) and bamboo (Acc. No. AEK66732.1). Color code: white, conserved; green, identical; cyan, similar and yellow, different residues. This alignment was generated using the SDSC Biology WorkBench [42].

In the perennial ryegrass mitochondrial genome, the *rps*4-p and *rps*4 genes are conserved to each other in the 5^′^ end (599 nucleotides with a 99% sequence identity) but they do not share the sequence. The BLASTn result confirmed that the 3^′^ end of the *rps*4-p gene has no homology with *rps*4 gene of other species reported so far. Thus, the *rps*4-p gene might be a variant of the ribosomal gene, *rps*4 or a pseudogene in the perennial ryegrass mitochondrial genome. The amino acid sequences of *rps*4 and *rps*4-p genes in perennial ryegrass were aligned with the amino acid sequences of the *rps*4 gene of maize (Acc. No. YP_588274.1), sorghum (Acc. No. YP_762343.1), rice (Acc. No. YP_514660.1), wheat (Acc. No. ADE08097.1) and bamboo (Acc. No. AEK66732.1), (Figure 3). The alignment showed that only 196 amino acids (45%) of the *rps*4-p gene are conserve to the *rps*4 gene. Transcriptome analysis confirmed that both *rps*4 and *rps*4-p are expressed in the reproductive tissue of the perennial ryegrass. Both *rps*4 and *rps*4-p genes had low normalized expression pattern (<1,000 reads per kbp length) (Table 7). In addition, we also found two ribosomal protein genes, *rps*3 and *rpl*16, sharing 110 bp of sequence between them (Figure 1, Table 4). The shared sequence was located in the second exon of *rps*3 (*rps*3b) and at the beginning of the *rpl*16 gene. Similarly, in the wheat KS3-type mitochondrial genome, *KSorf*1484 has 46 bp shared sequence with the *cob* gene [41].

Plant mitochondrial genomes contain TEs, DNA sequences that can move from one position to another. TEs can constitute an appreciable fraction in the genome and are found in most species with the exception of liverwort [33]. In the perennial ryegrass mitochondrial genome, we found 22 and 95 TE fragments of various sizes covering a total of 3,008 and 22,545 bp based on comparison to the Triticeae and Poaceae databases, respectively (Table 6). The sequences varied in length and the elements were dispersed in the perennial ryegrass mitochondrial genome (Additional file 5: Table S5). The TEs appear to be more abundant in grasses than cereals. In the perennial ryegrass mitochondrial genome only 0.44% or 3.32% of the genome showed homology with transposable like elements, most of which resemble retrotransposable elements, suggesting that their contribution to the expanded perennial ryegrass mitochondrial genome is negligible.

### Gene expression profile

In this study, the expression profile of perennial ryegrass mitochondrial genes was studied in reproductive flower tissues (Table 7). In reproductive tissues, the mitochondrial density and activity is especially high presumably because the energy and biosynthetic requirement is particularly high during reproduction, i.e., during pollen development [43,44]. The generally high expression level of all of the encoded subunits of the respiratory complexes in the mitochondrial genome (Table 7) is consistent with that observation. However, there were some variations between the expression levels of the complexes:

Complex I, the NADH dehydrogenase, contains around 40 subunits, one copy each per complex, in higher plants [45] where nine are mitochondrially encoded in perennial ryegrass (Table 4). The normalized expression levels for all of the complex I subunits were generally high and varied about 3-fold (Table 7).

Complex IV, cytochrome *c* oxidase, contains 12–13 subunits out of which three are mitochondrially encoded in most plants including perennial ryegrass (Table 4). All three subunits were highly expressed (Table 7).

Complex V, the ATP synthase, consists of about 15 subunits, five of which are encoded by the mitochondrial genome in perennial ryegrass (Table 4), while the remaining are encoded in the nucleus, synthesized on free ribosomes in the cytosol and imported into the mitochondria to be assembled with the mitochondrially encoded subunits into a complex in the inner membrane. The normalized expression levels of all the ATP synthase (complex V) genes was relatively high in perennial ryegrass reproductive tissues and varied <3-fold with the exception of *atp*9 (encoding subunit *c* of the Fo), which had a 2.5 times higher expression than the second highest, which is *atp*1 (encoding subunit alpha of the F1) (Table 7). This may be significant given that subunit *c* of the complex is present in 10–15 copies per complex and the alpha subunit is present in three copies while all the other mitochondrially encoded subunits are only present in one copy each per complex V [46]. Thus, the mRNA levels in perennial ryegrass mitochondria as expressed by the normalized read numbers are correlated with the biosynthetic requirement for complex V subunits. Previous studies have shown that the *atp*1 gene is highly expressed in male flower of date palm, and in pollen mother cells of *Arabidopsis*[28,47].

Finally, the four genes encoding proteins involved in cytochrome *c* biosynthesis, *ccm*B, *ccm*C, *ccm*FC and *ccm*FN, were all expressed, but not at particularly high levels (Table 7). An earlier microarray analysis of mitochondrial gene expression at the early stage of wheat shoot tissues reported that the *ccm*FN gene showed increased transcript level under three different stress conditions, low temperature (4°C), high salinity (0.2 M NaCl) and high osmotic potential (0.3 M mannitol) [48].

Protein biosynthesis in plant mitochondria takes place on bacterial-type ribosomes, where 14 of the subunits are encoded in the perennial ryegrass genome (Table 4). Out of these, eight had low normalized expression levels (<1,000 reads per kbp length), while three (*rps*3, *rps*12 and *rpl*16) showed high expression levels (>5,000 reads per kb length) (Table 7). Four of the ribosomal protein genes were hardly expressed at all (*rps*14-1, *rps*14-2, *rpl*5-1 and *rpl*5-2) which may be because they are non-functional, or because they are required in other tissues, but not in the reproductive tissues in perennial ryegrass. Consistent with the latter hypothesis, two ribosomal protein genes, *rps*1 and *rps*19, are much more abundantly expressed in roots than in other tissues of date palm [28].

Transcription does not necessarily lead to protein synthesis. An astonishing 48.5% and 30.8% of the total mitochondrial genomes of rice and date palm, respectively, are transcribed, which is due to RNA synthesis from large parts of the regions outside the annotated genes. For comparison, only 6.5% of the date palm mitochondrial genome is translated into proteins [28,37]. The functions of the transcribed inter-genic regions of plant mitochondrial genomes are not well understood.

The expression of the mitochondrial genes in plants is carried out by phage-type RNA polymerase encoded in the nuclear genome [49]. The process of gene expression in plant mitochondrial DNA is rather complex, influenced by multiple promoters, RNA processing and particularly at the post-transcriptional processes, splicing and editing [50-52]. Prior to transcription, the RNA polymerase is capable of promoter recognition, initiation, and elongation on their own but need auxillary factors to recognize all transcription initiation sites [53]. The sequence analysis of the *Arabidopsis* mitochondrial genome showed that potential promoter motifs exist in the inter-generic regions [54]. In addition, a number of annotated genes do not show potential promoter sequences confirming the possibility that other sequences can initiate transcription [55]. For this reason, transcription is actually initiated from a variety of promoter sites in the genome [50,55]. Thus, transcription in plant mitochondria produces both cryptic transcripts from regions that do not contain genes or from the opposite DNA strand of the genes; as well as defective transcripts encoded by the genes but failing to complete the complex post-transcriptional process to become functional transcripts [56]. Once initiated, transcription sometimes give rise to extremely large transcripts due to the absence of efficient transcription termination in plant mitochondria [57]. This contributes significantly to the transcription of the inter-genic regions. Therefore, large portions of the mitochondrial genome are transcribed but not translated into proteins.

## Conclusions

For the first time, the mitochondrial genome of perennial ryegrass has been sequenced, successfully assembled and annotated. The data presented here constitute a primary platform to understand the organization and function of the mitochondrial genome in one of the most important forage and turf grass species. The circular mitochondrial master molecule will be useful for comparative mitochondrial genomics and for future research on agronomically important traits such as CMS.

### Perspectives

Perennial ryegrass is a dominant forage species in the temperate regions worldwide, and its main role is to provide forage to the ruminant animals. Eighty per cent of the world’s cow milk and 70% of the world’s beef and veal are produced from temperate grasslands [58]. A major portion of these grasslands is covered by perennial ryegrass, which is, however, not well adapted to regions with severe winter or hot summer [58], unless the geographic range of the species can be extended by developing more robust cultivars. One way to increase productivity, nutritional quality and tolerance towards biotic and abiotic stress is to maximize the genetically available heterosis using hybrid breeding schemes. However, hybrid seed production requires a tool to efficiently control pollination, a tool such as CMS. The mitochondrial genome is a key to understanding the origin and function of CMS and will – in the long term – facilitate the development of hybrid cultivars in allogamous forage grasses.

## Methods

### Plant material

The perennial ryegrass genotype F1-30 was used for mitochondrial genome sequencing. F1-30 was developed from a cross between a genotype of the Italian cultivar Veyo and the Danish ecotype Falster [59]. The F1-30 genotype was multiplied by clonal propagation and grown in 15 cm × 15 cm plastic pots in the greenhouse in order to develop plant material for mitochondrial DNA (mtDNA) extraction.

### Isolation of intact mitochondria

The plants were kept in darkness for 24 h prior to mitochondrial isolation in order to reduce the amount of starch in the chloroplasts. For each batch of mtDNA isolation a total of 30 g young leaves were collected from 4-month-old clones of F1-30 to isolate intact mitochondria. All the equipment and buffers were kept at 4°C before the extraction, and all the steps were conducted on ice or at 4°C.

The leaves were cut into small pieces (5–10 mm) with scissors. Thirty g leaf pieces were homogenized for 1 min in 300 ml extraction buffer containing 0.3 M mannitol, 5 mM EDTA, 30 mM MOPS (pH 7.3 adjusted with 1 M KOH), using a chilled mortar and pestle. The reagents 0.2% (w/v) BSA, 5 mM DTT and 1% (w/v) PVPP, were added to the extraction buffer prior to use. The crude homogenate was filtered through two layers of cotton cloth followed by centrifugation at 2,000 *g* for 10 min to pellet starch, nuclei and chloroplast. The recovered supernatant was centrifuged at 10,000 *g* for 15 min to pellet intact mitochondria.

The mitochondrial pellet was resuspended in 7 ml DNAse-I buffer containing 0.44 M sucrose, 50 mM Tris–HCl (pH 8.0) and 10 mM MgCl_2_. Eight mg of DNAse-I recombinant, grade I (Roche, Mannheim, Germany) was dissolved in 1 ml DNAse-I buffer, and added to the mitochondrial suspension to give a final concentration of 1 mg/ml DNAse-I. Digestion was allowed to continue on ice for 2 h to degrade any nuclear and chloroplast DNA present outside the mitochondria. The digestion was terminated by adding 0.5 M EDTA (pH 8.0) to a final concentration of 25 mM. Mitochondria were re-pelleted at 16,000 *g* for 10 min. The pellet was resuspended in 25 ml of wash buffer (0.3 M mannitol, 1 mM EDTA, 10 mM MOPS, pH 7.2 adjusted with 1 M KOH). Intact mitochondria were washed twice by resuspension in wash buffer and re-pelleting at 16,000 *g* for 10 min.

### Extraction, purification and precipitation of mtDNA

The washed mitochondrial pellet was lysed by suspension in 2 ml lysis buffer containing 10 mM Tris–HCl (pH 8.0), 10 mM NaCl and 1 mM EDTA (pH 8.0), followed by the addition of 10% SDS to a final concentration of 1% (v/v) and incubation at 37°C for 30 min. DNA was purified according to the standard method [60] with slight modifications. An equal volume of phenol:chloroform (25:24 v/v) was added to the sample and centrifuged at 20,800 *g* for 15 min at room temperature. The aqueous phase was transferred to an eppendorf tube, and two additional cycles of phenol:chloroform extraction and two cycles of chloroform extraction were performed. The purified DNA was precipitated by adding 0.1 volume of 3 M sodium acetate (pH 5.5) and 2 volumes of cold (4°C) absolute ethanol (99.9%) to the purified DNA. The mixture was vortexed briefly and incubated at −20°C overnight. The precipitated DNA was recovered by centrifugation at 4°C at 20,375 *g* for 15 min. The ethanol was removed by decantation and 300 μl ice cold 70% (v/v) ethanol was added and centrifuged again at 4°C at 20,375 *g* for 5 min. The ethanol was removed and the pellet was air dried and resuspended in sterile Tris/EDTA buffer (pH 8.0) followed by an equal volume of R40 (40 μg/ml RNAse A (Roche, Mannheim, Germany) in Tris/EDTA buffer). The mtDNA was checked for quality by gel electrophoresis on a 1.5% agarose gel in 1× TAE buffer (Additional file 6: Figure 1). DNA from two batches of mtDNA isolation was pooled in order to obtain a sufficient amount for sequencing.

### Monitoring mitochondrial purity and intactness and protein concentration

During the mitochondrial preparation, we kept 1 ml samples from each preparation step (homogenate to supernatant) to be used for the determination of enzyme activation and protein concentration. The activity and latency of cytochrome *c* oxidase (CCO), an inner membrane enzyme, was measured at 550 nm in an assay medium containing 0.3 M sucrose, 50 mM Tris, 100 mM KCl and 45 μM reduced cytochrome *c*, pH adjusted to 7.2 using 1 M acetic acid plus or minus 0.05% (w/v) Triton X-100. The activity and latency of NAD^+^-dependent malate dehydrogenase (MDH), a matrix enzyme, was measured at 340 nm in the cuvette using 1 ml assay medium containing 0.3 M sucrose, 20 mM MOPS-KOH, pH 7.0, 20 μl of 100 mM oxaloacetate, pH 7.0, 5 μl of 200 mM salicylhydroxamic acid, 2 μl of 0.2 mM antimycin and 4 μl of 50 mM NADH plus or minus 0.05% (w/v) Triton X-100. In both cases the enzyme latency was calculated as,

$$\begin{array}{l} \text{Percentage}\;\text{intact}=([(\text{rate}+\text{Triton})\\ \;-(\text{rate}-\text{Triton})]/(\text{rate}+\text{Triton}))\\ \;\times 100\% \end{array}$$

[14]. The latency of CCO activity is a measure of the integrity of mitochondrial outer membrane as the substrate, reduced cytochrome *c*, can not penetrate an intact outer membrane to reach the active site on the outer surface of the inner membrane. The latency of MDH activity is a measure of the integrity of the mitochondrial inner membrane as NADH can not cross an intact inner membrane to reach the enzyme present in the mitochondrial matrix [14,15].

The protein concentration in the various fractions was measured at 562 nm using Bicinchoninic acid (BCA) protein assay kit (Sigma) containing BCA working reagent, BSA protein standard and 5% deoxycholic acid as recommended by the manufacturer.

### Library preparation and next-generation sequencing

A sequencing library for F1-30 was prepared according to the Rapid Library Preparation Method Manual October 2009 (Rev. Jan 2010) using 500 ng mtDNA. Sequencing was performed on a Roche 454 GS-FLX Titanium instrument (software version 2.3) following the manufacturer’s recommendations.

### *De novo* assembly of the mitochondrial genome

Adaptor removal, quality filtering (quality score 99.8%), and reference assembly against the chloroplast genome was performed using the CLC Genomics Workbench software (v.5.0). The chloroplast reads were removed by performing a reference assembly against the perennial ryegrass chloroplast genome (GenBank Acc. No.: NC_009950.1) using the parameters: similarity, 0.98; conflict resolution, vote (A, C, G, T); non-specific matches, random and masking of references, none. Reads mapping to the chloroplast genome were subsequently removed from the dataset. The sequence reads were assembled into contigs using the CLC Genomics Workbench software (v.5.0) with the parameters: Mismatch cost, 2; Insertion cost, 3; Deletion cost, 3; Length fraction, 0.5; Similarity fraction, 0.99. Gap closure and manual inspection and editing were performed using the SeqMan software (v.5.0.3). Mitochondrial genome contigs were identified by BLASTn (E-value 1×10^−10^). The PipMaker software [20] was used to identify repetitive regions within and among the mitochondrial contigs in order to facilitate primer design. DNASIS Max (v.2.9.) was used to blast in-house against the perennial ryegrass genome scaffolds in order to validate the order of the contigs in the mitochondrial genome. Primers were designed using the Primer3 software (v.0.4.0). Genomic DNA of F1-30 was used as template for PCR to amplify the gaps between contigs. The purified PCR products were sequenced by Eurofins MWG Operon (Ebersberg, Germany). The gap sequences were incorporated into the assembly using the SeqMan software.

### Genome annotation and analysis

Annotation of the mitochondrial genome was done using the Maker2 pipeline [61]. In a first round of analysis we used an in-house assembly of the F1-30 transcriptome (unpublished) as initial evidence for gene prediction. We also utilized a collection of plant mitochondrial protein sequences from various organisms included in the genome annotation software package Mitofy for plant mitochondria (http://dogma.ccbb.utexas.edu/mitofy). Repeat masking was performed with a grass-specific repeat database from RepBase [62]. After an initial round of gene prediction, a training file was generated for the *ab initio* gene predictor SNAP, and an additional round of gene prediction was performed. The resulting GFF3 file was loaded into Apollo [63] for visualization and manual curation of the genes after taking all the available evidence into account. Structural RNA genes were identified using tRNAscan-SE 1.21 (for tRNAs) and RNAmmer 1.2 for rRNAs [64,65]. Search for ORFs was performed using CLC Genomics Workbench (v.5.0).

Sequence repeats were investigated using PipMaker with default parameter settings [20]. SIR were detected using the inverted repeat finder software [66] (match 2; mismatch 3; delta 5; match probability 80; indel probability 10; minimum alignment score 40; maximum length to report 100,000; maximum loop 100,000; maximum loop separation for tuple of length 4). Tandem repeats were detected using the Tandem Repeat Finder (v.4.04) developed by [67] with parameters (alignment parameters [match, mismatch, indel; 2,7,7], min. align. score 50; max. period size 2,000). SSRs were identified using the msatcommander 0.8.2-WINXP.Zip software package [68]. The parameters for SSR detection were 1- to 2-nucleotide (nt) repeats of at least 10 nt length and 3- to 6-nt repeats with at least three repeat units (Additional file 4: Table S4).

TEs were identified using CENSOR (with default parameter settings, using Poaceae and Triticeae as reference [62].

### RNA preparation and sequencing

For transcriptome analysis, pollen and stigma tissue samples were collected from the F1-30 genotype grown under standard growing conditions in a greenhouse. A sealed paper bag was put over the inflorescences for 8 hours at anthesis to collect the pollen. The pollen was harvested after 8 hours, frozen in liquid nitrogen and stored at –80°C. Unpollinated stigmas were isolated from flowers just before anthesis, frozen in liquid nitrogen and stored at –80°C. Total RNA was extracted from each sample using the RNeasy™ Plant Mini Kit following the manufactures instructions (Qiagen, Valencia, CA, USA ), and the RNA integrity was measured with a RNA 6000 Nano Labchip™ on the Agilent 2100 Bioanalyzer™ (Agilent Technologies, Santa Clara, CA, USA). Samples were sequenced on an Illumina HiSeq2000 system.

### Read quality and trimming of sequences

Using the program FastQC (Babraham Institute, CA, USA) we were able to visualize the read quality and length of the Illumina raw reads. Using the output from this program we determined that Illumina adaptors were present at the 3^′^ end of the reads. It also indicated to us that the paired-end reads were overlapping. Using this to our advantage, we used the program fastq-join.pl [http://code.google.com/p/ea-utils] to merge reads with an overlap of 10 bp after removing the Illumina adaptors on the 3^′^ end of the read using the program Homer-Tools [69].

### Transcriptome analyses

Illumina 101 bp reads from reproductive tissue samples were used for gene expression analysis of the 39 protein-coding genes. Reads were mapped onto the sequences of the 39 genes using Bowtie [70], allowing a maximum of 2 mismatches in the first 25 bp. The program RSEM [71] was used to calculate RNA-seq read abundance from the SAM alignments. The expression of each gene was calculated by dividing the abundance estimates from RSEM by the length of the gene (kbp).

## Competing interests

The authors declare that they have no competing interests.

## Authors’ contributions

MSI, BS and TA designed the experiment. MSI and IMM developed the mtDNA extraction protocol and measured the intactness of mitochondria in the crude mitochondrial preparations. CB and FP constructed the genome sequence library of the mtDNA and performed the Roche 454 GS-FLX Titanium sequencing. TA performed the *de novo* assembly of the mitochondrial genome, MSI and BS designed primers. MSI completed the final assembly of the mitochondrial genome in consultation with BS and TA. SLB annotated the mitochondrial genome; MSI and TA finalized the genome annotation. JDF performed the transcript data analysis. MSI drafted the manuscript, which was improved by IMM, BS, SLB and TA. All authors read and approved the final manuscript.

## Supplementary Material

Additional file 1: Table S1 Open reading frames (ORFs) in the perennial ryegrass mitochondrial genome. ORFs greater than 300 nucleotides and located outside the identified genes were included in the list. ORFs beginning with a methionine codon (ATG) and end with a termination codon were considered. Stop codon is included in the ORF length. The 149 ORFs were numbered ORF_1 to ORF_149. ^a^Plus and minus, encoded by the forward and reverse DNA strand, respectively. Click here for file

Additional file 2: Table S2 Short inverted repeats in the perennial ryegrass mitochondrial genome. Click here for file

Additional file 3: Table S3 Tandem repeat content in the perennial ryegrass mitochondrial genome. Indices of the repeat relative to the start of the sequence, number of copies aligned with the consensus pattern, size of consensus pattern (may differ from the repeat size), percent of matches between adjacent copies overall, and percent of indels between adjacent copies overall. Click here for file

Additional file 4: Table S4 SSRs found in the perennial ryegrass mitochondrial genome. ^a^Plus and minus, encoded by the forward and reverse DNA strand, respectively; ^b^plus and minus, present and absent, respectively; ^c^lower case letter indicates the exon; RC= Reverse complement. Click here for file

Additional file 5: Table S5 Transposable elements in the perennial ryegrass mitochondrial genome. Transposable elements derived from Triticeae (**A**) and Poaceae (**B**). Left and right, position of the transposable element in the mitochondrial genome (From/To indicates start/end of positions of the transposable elements. Orientation: d, direct; c, complementary. Sim indicates value of similarity between 2 aligned fragments; Pos is the ratio of positives to alignment length; Mm:Ts is a ratio of mismatches to transitions in the nucleotide alignment. Score, alignment score obtained from blast. Click here for file

Additional file 6: Figure 1 (A-E) Checking of nuclear DNA contamination in isolated perennial ryegrass mtDNA. A-E: Lane 1, 100 bp DNA ladder; 2, F1-30 mtDNA; 3, F1-30 genomic DNA and 4–5, genomic DNA of two other genotypes of perennial ryegrass. DNA amplified by five SSR primer sets, G03_075, G03_044, G05_070, G05_071 [72] and LpSSR006 [73], selected from different linkage groups of perennial ryegrass. Click here for file
