# Supplementary material for: The genome and transcriptome of perennial ryegrass mitochondria
Source: BMC Genomics. 2013 Mar 23;14:202. doi: 10.1186/1471-2164-14-202 (PMC3664089; doi:10.1186/1471-2164-14-202)
Supplement: Additional file 1: Table S1 — Open reading frames (ORFs) in the perennial ryegrass mitochondrial genome. ORFs greater than 300 nucleotides and located outside the identified genes were included in the list. ORFs beginning with a methionine codon (ATG) and end with a termination codon were considered. Stop codon is included in the ORF length. The 149 ORFs were numbered ORF_1 to ORF_149. aPlus and minus, encoded by the forward and reverse DNA strand, respectively. [file 1471-2164-14-202-S1.docx]

**Additional file 2: Supplementary Table S1- Open reading frames (ORFs) in the perennial ryegrass mitochondrial genome.**

| **ORFs_ID** | **Position** | | **Length** | **^a^DNA strand** |
| --- | --- | --- | --- | --- |
|  | **From** | **To** |  |  |
| ORF_1 | 10540 | 11661 | 1122 | + |
| ORF_2 | 12406 | 12774 | 369 | + |
| ORF_3 | 13841 | 14149 | 309 | - |
| ORF_4 | 23767 | 24159 | 393 | + |
| ORF_5 | 24807 | 25307 | 501 | - |
| ORF_6 | 26244 | 26564 | 321 | - |
| ORF_7 | 27381 | 28364 | 984 | + |
| ORF_8 | 35899 | 36225 | 327 | + |
| ORF_9 | 55771 | 56466 | 696 | + |
| ORF_10 | 58671 | 59276 | 606 | - |
| ORF_11 | 60462 | 60791 | 330 | - |
| ORF_12 | 71370 | 71735 | 366 | + |
| ORF_13 | 74257 | 74622 | 366 | - |
| ORF_14 | 75034 | 75492 | 459 | - |
| ORF_15 | 75526 | 75972 | 447 | - |
| ORF_16 | 80629 | 81078 | 450 | - |
| ORF_17 | 87387 | 87779 | 393 | + |
| ORF_18 | 88809 | 89393 | 585 | - |
| ORF_19 | 91410 | 91748 | 339 | - |
| ORF_20 | 110681 | 111190 | 510 | + |
| ORF_21 | 114156 | 115187 | 1032 | - |
| ORF_22 | 125723 | 126154 | 432 | - |
| ORF_23 | 127047 | 127442 | 396 | + |
| ORF_24 | 127934 | 128254 | 321 | - |
| ORF_25 | 128806 | 129285 | 480 | + |
| ORF_26 | 129939 | 130280 | 342 | - |
| ORF_27 | 133688 | 134068 | 381 | + |
| ORF_28 | 138573 | 138875 | 303 | + |
| ORF_29 | 140656 | 141069 | 414 | - |
| ORF_30 | 141953 | 142336 | 384 | + |
| ORF_31 | 143484 | 143870 | 387 | + |
| ORF_32 | 158509 | 158811 | 303 | - |
| ORF_33 | 166011 | 166379 | 369 | - |
| ORF_34 | 183529 | 183855 | 327 | + |
| ORF_35 | 183834 | 184181 | 348 | - |
| ORF_36 | 191722 | 192351 | 630 | + |
| ORF_37 | 192204 | 194171 | 1968 | + |
| ORF_38 | 194604 | 194993 | 390 | + |
| ORF_39 | 208669 | 208974 | 306 | + |
| ORF_40 | 210241 | 210597 | 357 | + |
| ORF_41 | 226527 | 226907 | 381 | + |
| ORF_42 | 227502 | 227993 | 492 | - |
| ORF_43 | 227739 | 228056 | 318 | + |
| ORF_44 | 253894 | 254214 | 321 | - |
| ORF_45 | 264696 | 265082 | 387 | - |
| ORF_46 | 266777 | 267115 | 339 | - |
| ORF_47 | 266802 | 267164 | 363 | + |
| ORF_48 | 267090 | 267512 | 423 | - |
| ORF_49 | 267334 | 267639 | 306 | - |
| ORF_50 | 270646 | 270972 | 327 | - |
| ORF_51 | 272920 | 273231 | 312 | - |
| ORF_52 | 275270 | 275587 | 318 | - |
| ORF_53 | 278978 | 279331 | 354 | + |
| ORF_54 | 279054 | 279422 | 369 | + |
| ORF_55 | 281712 | 282035 | 324 | + |
| ORF_56 | 281933 | 282289 | 357 | - |
| ORF_57 | 285296 | 285673 | 378 | - |
| ORF_58 | 293736 | 294068 | 333 | + |
| ORF_59 | 297324 | 297755 | 432 | - |
| ORF_60 | 305046 | 305543 | 498 | - |
| ORF_61 | 305152 | 305565 | 414 | + |
| ORF_62 | 306613 | 306915 | 303 | + |
| ORF_63 | 314205 | 314756 | 552 | + |
| ORF_64 | 315446 | 315865 | 420 | + |
| ORF_65 | 315905 | 318475 | 2571 | + |
| ORF_66 | 317000 | 317311 | 312 | - |
| ORF_67 | 319063 | 319506 | 444 | - |
| ORF_68 | 326054 | 326398 | 345 | + |
| ORF_69 | 337840 | 338181 | 342 | + |
| ORF_70 | 338835 | 339314 | 480 | - |
| ORF_71 | 339866 | 340186 | 321 | + |
| ORF_72 | 340678 | 341073 | 396 | - |
| ORF_73 | 341966 | 342397 | 432 | + |
| ORF_74 | 352929 | 353330 | 402 | + |
| ORF_75 | 353314 | 353961 | 648 | + |
| ORF_76 | 356929 | 357438 | 510 | - |
| ORF_77 | 363555 | 363893 | 339 | + |
| ORF_78 | 372182 | 372526 | 345 | + |
| ORF_79 | 375070 | 375465 | 396 | + |
| ORF_80 | 375541 | 375987 | 447 | + |
| ORF_81 | 392153 | 392521 | 369 | + |
| ORF_82 | 399721 | 400023 | 303 | + |
| ORF_83 | 414638 | 415003 | 366 | + |
| ORF_84 | 418995 | 419351 | 357 | - |
| ORF_85 | 422756 | 423058 | 303 | - |
| ORF_86 | 426215 | 426694 | 480 | + |
| ORF_87 | 431389 | 431925 | 537 | - |
| ORF_88 | 432243 | 432578 | 336 | - |
| ORF_89 | 433827 | 434315 | 489 | - |
| ORF_90 | 435094 | 435411 | 318 | - |
| ORF_91 | 435157 | 435648 | 492 | + |
| ORF_92 | 436244 | 436624 | 381 | - |
| ORF_93 | 452562 | 452918 | 357 | - |
| ORF_94 | 454185 | 454490 | 306 | - |
| ORF_95 | 468166 | 468555 | 390 | - |
| ORF_96 | 468988 | 470955 | 1968 | - |
| ORF_97 | 470808 | 471437 | 630 | - |
| ORF_98 | 478978 | 479325 | 348 | + |
| ORF_99 | 479304 | 479630 | 327 | - |
| ORF_100 | 488675 | 489160 | 486 | - |
| ORF_101 | 489992 | 490486 | 495 | - |
| ORF_102 | 490549 | 490857 | 309 | - |
| ORF_103 | 492286 | 492612 | 327 | - |
| ORF_104 | 492901 | 493356 | 456 | + |
| ORF_105 | 493638 | 493952 | 315 | - |
| ORF_106 | 498051 | 498383 | 333 | - |
| ORF_107 | 508045 | 508494 | 450 | - |
| ORF_108 | 508915 | 509352 | 438 | - |
| ORF_109 | 510125 | 510427 | 303 | + |
| ORF_110 | 512572 | 512946 | 375 | - |
| ORF_111 | 514123 | 514437 | 315 | - |
| ORF_112 | 516952 | 517356 | 405 | - |
| ORF_113 | 517459 | 517890 | 432 | - |
| ORF_114 | 518510 | 518815 | 306 | - |
| ORF_115 | 529241 | 529630 | 390 | - |
| ORF_116 | 533711 | 534133 | 423 | + |
| ORF_117 | 537970 | 538356 | 387 | + |
| ORF_118 | 544491 | 544823 | 333 | + |
| ORF_119 | 545895 | 546344 | 450 | + |
| ORF_120 | 551027 | 551344 | 318 | - |
| ORF_121 | 555301 | 555645 | 345 | + |
| ORF_122 | 555988 | 556377 | 390 | - |
| ORF_123 | 556967 | 557386 | 420 | + |
| ORF_124 | 557316 | 557621 | 306 | - |
| ORF_125 | 581216 | 581518 | 303 | + |
| ORF_126 | 587889 | 588254 | 366 | - |
| ORF_127 | 619944 | 620318 | 375 | - |
| ORF_128 | 623730 | 624281 | 552 | - |
| ORF_129 | 627066 | 627425 | 360 | + |
| ORF_130 | 628115 | 628468 | 354 | - |
| ORF_131 | 628260 | 628658 | 399 | - |
| ORF_132 | 632226 | 632549 | 324 | + |
| ORF_133 | 632944 | 633246 | 303 | + |
| ORF_134 | 640411 | 640716 | 306 | + |
| ORF_135 | 641399 | 642124 | 726 | + |
| ORF_136 | 646578 | 646973 | 396 | + |
| ORF_137 | 648164 | 648526 | 363 | - |
| ORF_138 | 649734 | 650147 | 414 | - |
| ORF_139 | 650170 | 650508 | 339 | - |
| ORF_140 | 650716 | 651612 | 897 | - |
| ORF_141 | 651240 | 651551 | 312 | - |
| ORF_142 | 656187 | 656585 | 399 | + |
| ORF_143 | 664528 | 665346 | 819 | + |
| ORF_144 | 666701 | 667135 | 435 | - |
| ORF_145 | 667191 | 667793 | 603 | - |
| ORF_146 | 668199 | 668528 | 330 | - |
| ORF_147 | 668709 | 669062 | 354 | - |
| ORF_148 | 675717 | 676295 | 579 | - |
| ORF_149 | 677755 | 678498 | 744 | + |
