# Supplementary material for: The genome and transcriptome of perennial ryegrass mitochondria
Source: BMC Genomics. 2013 Mar 23;14:202. doi: 10.1186/1471-2164-14-202 (PMC3664089; doi:10.1186/1471-2164-14-202)
Supplement: Additional file 2: Table S2 — Short inverted repeats in the perennial ryegrass mitochondrial genome. [file 1471-2164-14-202-S2.docx]

**Additional file 3: Supplementary Table S2 – Short inverted repeats in the perennial ryegrass mitochondrial genome.**

| Left indices | | Length | Right indices | | Length | Loop | Percent | Percent | Score |
| --- | --- | --- | --- | --- | --- | --- | --- | --- | --- |
| From | To |  | From | To |  |  | match | indel |  |
| 24,403 | 24,471 | 69 | 56,891 | 56,959 | 69 | 32,419 | 81.15 | 0.00 | 73 |
| 45,325 | 45,358 | 34 | 76,216 | 76,249 | 34 | 30,857 | 91.43 | 5.71 | 51 |
| 35,921 | 35,968 | 48 | 76,647 | 76,693 | 47 | 40,678 | 87.75 | 6.12 | 62 |
| 45,321 | 45,374 | 54 | 76,855 | 76,909 | 55 | 31,480 | 87.27 | 1.82 | 73 |
| 79,673 | 79,723 | 51 | 82,483 | 82,530 | 48 | 2,759 | 80.77 | 9.61 | 44 |
| 35,357 | 35,432 | 76 | 119,240 | 119,304 | 65 | 83,807 | 82.89 | 14.47 | 65 |
| 66,837 | 66,861 | 25 | 126,017 | 126,041 | 25 | 59,155 | 96.00 | 0.00 | 45 |
| 119,348 | 119,415 | 68 | 137,949 | 138,017 | 69 | 18,533 | 94.20 | 1.45 | 116 |
| 79,667 | 79,718 | 52 | 138,450 | 138,506 | 57 | 58,731 | 84.48 | 12.07 | 57 |
| 119,348 | 119,412 | 65 | 139,226 | 139,291 | 66 | 19,813 | 95.45 | 1.51 | 115 |
| 76,213 | 76,253 | 41 | 139,954 | 139,994 | 41 | 63,700 | 82.92 | 0.00 | 47 |
| 119,342 | 119,412 | 71 | 148,711 | 148,780 | 70 | 29,298 | 78.08 | 6.85 | 56 |
| 96,901 | 96,926 | 26 | 161,686 | 161,711 | 26 | 64,759 | 92.30 | 0.00 | 42 |
| 144,270 | 144,312 | 43 | 164,653 | 164,695 | 43 | 20,340 | 79.07 | 0.00 | 41 |
| 149,071 | 149,103 | 33 | 175,211 | 175,243 | 33 | 26,107 | 100.00 | 0.00 | 66 |
| 148,747 | 148,778 | 32 | 175,485 | 175,516 | 32 | 26,706 | 100.00 | 0.00 | 64 |
| 118,963 | 118,987 | 25 | 177,596 | 177,620 | 25 | 58,608 | 92.00 | 0.00 | 40 |
| 121,678 | 121,710 | 33 | 182,433 | 182,466 | 34 | 60,722 | 91.17 | 2.94 | 51 |
| 134,603 | 134,641 | 39 | 196,431 | 196,469 | 39 | 61,789 | 100.00 | 0.00 | 78 |
| 181,682 | 181,705 | 24 | 200,226 | 200,249 | 24 | 18,520 | 95.83 | 0.00 | 43 |
| 178,488 | 178,525 | 38 | 201,129 | 201,166 | 38 | 22,603 | 89.47 | 0.00 | 56 |
| 218,698 | 218,722 | 25 | 224,349 | 224,373 | 25 | 5,626 | 100.00 | 0.00 | 50 |
| 209,978 | 210,007 | 30 | 226,033 | 226,063 | 31 | 16,025 | 93.50 | 3.23 | 50 |
| 214,827 | 214,859 | 33 | 236,474 | 236,506 | 33 | 21,614 | 87.80 | 0.00 | 46 |
| 229,385 | 229,453 | 69 | 237,808 | 237,876 | 69 | 8,354 | 76.81 | 0.00 | 58 |
| 216,149 | 216,194 | 46 | 237,811 | 237,856 | 46 | 1,616 | 78.20 | 0.00 | 42 |
| 206,581 | 206,637 | 57 | 249,013 | 249,069 | 57 | 42,375 | 85.96 | 0.00 | 74 |
| 184,280 | 184,305 | 26 | 256,662 | 256,689 | 28 | 72,356 | 92.86 | 7.14 | 42 |
| 221,730 | 221,775 | 46 | 262,952 | 262,998 | 47 | 41,176 | 80.85 | 2.13 | 47 |
| 192,546 | 192,715 | 170 | 268,346 | 268,511 | 166 | 75,630 | 72.51 | 3.51 | 95 |
| 229,508 | 229,556 | 49 | 277,089 | 277,133 | 45 | 47,532 | 84.00 | 12.00 | 48 |
| 277,078 | 277,132 | 55 | 282,580 | 282,634 | 55 | 5,447 | 98.18 | 0.00 | 105 |
| 270,459 | 270,501 | 43 | 292,187 | 292,229 | 43 | 21,685 | 90.69 | 0.00 | 66 |
| 263,486 | 263,517 | 32 | 301,194 | 301,225 | 32 | 37,676 | 100.00 | 0.00 | 64 |
| 213,279 | 213,316 | 38 | 301,940 | 301,977 | 38 | 88,623 | 89.47 | 0.00 | 56 |
| 231,569 | 231,632 | 64 | 307,419 | 307,477 | 59 | 75,786 | 87.50 | 7.81 | 78 |
| 224,319 | 224,344 | 26 | 307,624 | 307,649 | 26 | 83,279 | 92.31 | 0.00 | 42 |
| 301,997 | 302,029 | 33 | 307,874 | 307,906 | 33 | 5,844 | 87.87 | 0.00 | 46 |
| 268,097 | 268,118 | 22 | 308,384 | 308,405 | 22 | 40,265 | 100.00 | 0.00 | 44 |
| 292,273 | 292,364 | 92 | 328,110 | 328,205 | 96 | 35,745 | 94.79 | 4.16 | 159 |
| 282,583 | 282,632 | 50 | 328,300 | 328,349 | 50 | 45,667 | 98.00 | 0.00 | 95 |
| 229,508 | 229,556 | 49 | 328,309 | 328,352 | 44 | 98,752 | 81.63 | 10.20 | 43 |
| 291,691 | 291,721 | 31 | 329,521 | 329,551 | 31 | 37,799 | 96.77 | 0.00 | 57 |
| 307,292 | 307,322 | 31 | 332,113 | 332,143 | 31 | 24,790 | 90.32 | 0.00 | 47 |
| 316,166 | 316,260 | 95 | 336,485 | 336,579 | 95 | 20,224 | 71.87 | 2.08 | 53 |
| 317,330 | 317,511 | 182 | 339,047 | 339,228 | 182 | 21,535 | 68.82 | 4.30 | 66 |
| 328,300 | 328,356 | 57 | 348,701 | 348,757 | 57 | 20,344 | 98.24 | 0.00 | 109 |
| 277,064 | 277,135 | 72 | 348,703 | 348,773 | 71 | 71,567 | 97.22 | 1.38 | 132 |
| 328,278 | 328,310 | 33 | 348,766 | 348,798 | 33 | 20,455 | 90.91 | 0.00 | 51 |
| 348,708 | 348,749 | 42 | 350,725 | 350,772 | 48 | 1,975 | 83.33 | 12.50 | 44 |
| 282,580 | 282,624 | 45 | 350,725 | 350,775 | 51 | 68,100 | 82.35 | 11.76 | 45 |
| 299,327 | 299,352 | 26 | 352,087 | 352,112 | 26 | 52,734 | 92.31 | 0.00 | 42 |
| 361,801 | 361,829 | 29 | 365,759 | 365,787 | 29 | 3,929 | 96.55 | 0.00 | 53 |
| 349,134 | 349,158 | 25 | 380,913 | 380,937 | 25 | 31,754 | 92.00 | 0.00 | 40 |
| 371,533 | 371,552 | 20 | 383,503 | 383,522 | 20 | 11,950 | 100.00 | 0.00 | 40 |
| 365,667 | 365,707 | 41 | 391,221 | 391,264 | 44 | 25,513 | 86.36 | 6.82 | 52 |
| 301,290 | 301,312 | 23 | 393,111 | 393,133 | 23 | 91,798 | 95.65 | 0.00 | 41 |
| 319,132 | 319,158 | 27 | 405,465 | 405,491 | 27 | 86,306 | 96.29 | 0.00 | 49 |
| 383,290 | 383,322 | 33 | 409,429 | 409,461 | 33 | 26,106 | 100.00 | 0.00 | 66 |
| 383,017 | 383,048 | 32 | 409,754 | 409,785 | 32 | 26,705 | 100.00 | 0.00 | 64 |
| 348,708 | 348,779 | 72 | 409,752 | 409,821 | 70 | 60,972 | 79.45 | 5.47 | 63 |
| 391,266 | 391,285 | 20 | 414,313 | 414,332 | 20 | 23,027 | 100.00 | 0.00 | 40 |
| 372,149 | 372,298 | 150 | 433,595 | 433,752 | 158 | 61,296 | 76.39 | 8.69 | 104 |
| 371,495 | 371,541 | 47 | 441,383 | 441,428 | 46 | 69,841 | 80.85 | 2.12 | 47 |
| 415,881 | 415,942 | 62 | 443,188 | 443,247 | 60 | 27,245 | 95.16 | 3.22 | 105 |
| 438,783 | 438,807 | 25 | 444,437 | 444,461 | 25 | 5,629 | 100.00 | 0.00 | 50 |
| 437,090 | 437,120 | 31 | 453,152 | 453,181 | 30 | 16,031 | 93.55 | 3.22 | 50 |
| 444,139 | 444,190 | 52 | 461,914 | 461,965 | 52 | 17,723 | 100.00 | 0.00 | 104 |
| 380,008 | 380,045 | 38 | 461,993 | 462,030 | 38 | 81,947 | 89.47 | 0.00 | 56 |
| 424,962 | 425,144 | 183 | 470,059 | 470,241 | 183 | 44,914 | 72.43 | 2.16 | 107 |
| 462,910 | 462,933 | 24 | 481,454 | 481,477 | 24 | 18,520 | 95.83 | 0.00 | 43 |
| 461,993 | 462,030 | 38 | 484,634 | 484,671 | 38 | 22,603 | 89.47 | 0.00 | 56 |
| 454,305 | 454,370 | 66 | 493,024 | 493,089 | 66 | 38,653 | 74.63 | 2.98 | 45 |
| 467,870 | 467,917 | 48 | 501,597 | 501,645 | 49 | 33,679 | 91.83 | 2.04 | 76 |
| 502,179 | 502,219 | 41 | 507,463 | 507,503 | 41 | 5,243 | 95.12 | 0.00 | 72 |
| 502,179 | 502,203 | 25 | 513,617 | 513,641 | 25 | 11,413 | 92.00 | 0.00 | 40 |
| 430,942 | 430,970 | 29 | 515,946 | 515,974 | 29 | 84,975 | 100.00 | 0.00 | 58 |
| 537,069 | 537,103 | 35 | 537,497 | 537,530 | 34 | 393 | 86.11 | 8.33 | 41 |
| 539,081 | 539,118 | 38 | 539,730 | 539,766 | 37 | 611 | 84.61 | 7.69 | 42 |
| 473,451 | 473,783 | 333 | 543,634 | 543,966 | 333 | 69,850 | 82.79 | 5.83 | 351 |
| 540,234 | 540,291 | 58 | 544,937 | 544,994 | 58 | 4,645 | 94.82 | 0.00 | 101 |
| 537,487 | 537,526 | 40 | 545,851 | 545,889 | 39 | 8,324 | 90.00 | 2.50 | 58 |
| 480,796 | 480,826 | 31 | 555,600 | 555,629 | 30 | 74,773 | 90.62 | 9.37 | 43 |
| 504,579 | 504,618 | 40 | 557,752 | 557,793 | 42 | 53,133 | 95.24 | 4.76 | 70 |
| 507,463 | 507,503 | 41 | 570,192 | 570,232 | 41 | 62,688 | 95.12 | 0.00 | 72 |
| 557,752 | 557,793 | 42 | 572,591 | 572,630 | 40 | 14,797 | 95.23 | 4.76 | 70 |
| 556,499 | 556,532 | 34 | 573,365 | 573,400 | 36 | 16,832 | 86.11 | 5.55 | 43 |
| 548,983 | 549,077 | 95 | 579,109 | 579,209 | 101 | 30,031 | 75.25 | 5.94 | 65 |
| 539,159 | 539,192 | 34 | 629,956 | 629,989 | 34 | 90,763 | 91.17 | 0.00 | 53 |
| 591,026 | 591,071 | 46 | 636,279 | 636,321 | 43 | 45,207 | 80.43 | 6.52 | 41 |
| 593,091 | 593,142 | 52 | 636,279 | 636,330 | 52 | 43,136 | 82.69 | 0.00 | 59 |
| 605,674 | 605,711 | 38 | 649,423 | 649,460 | 38 | 43,711 | 89.47 | 0.00 | 56 |
| 622,148 | 622,254 | 107 | 651,672 | 651,778 | 107 | 29,417 | 68.80 | 3.67 | 40 |
| 603,541 | 603,565 | 25 | 661,322 | 661,346 | 25 | 57,756 | 96.00 | 0.00 | 45 |
