# Supplementary material for: The genome and transcriptome of perennial ryegrass mitochondria
Source: BMC Genomics. 2013 Mar 23;14:202. doi: 10.1186/1471-2164-14-202 (PMC3664089; doi:10.1186/1471-2164-14-202)
Supplement: Additional file 3: Table S3 — Tandem repeat content in the perennial ryegrass mitochondrial genome. Indices of the repeat relative to the start of the sequence, number of copies aligned with the consensus pattern, size of consensus pattern (may differ from the repeat size), percent of matches between adjacent copies overall, and percent of indels between adjacent copies overall. [file 1471-2164-14-202-S3.docx]

**Additional file 4: Supplementary Table S3 - Tandem repeat content in the perennial ryegrass mitochondrial genome.**

| Indices | | Repeat  size | Copy  number | Consensus  size | Percent  match | Percent  Indel | Alignment  score |
| --- | --- | --- | --- | --- | --- | --- | --- |
| From | To |  |  |  |  |  |  |
| 1,567 | 1,688 | 42 | 2.9 | 42 | 100 | 0 | 244 |
| 27,337 | 27,374 | 14 | 2.6 | 15 | 87 | 8 | 60 |
| 68,627 | 68,669 | 22 | 2 | 22 | 90 | 0 | 68 |
| 68,853 | 68,895 | 22 | 1.9 | 23 | 85 | 4 | 61 |
| 92,454 | 92,482 | 14 | 2.1 | 14 | 100 | 0 | 58 |
| 115,600 | 115,624 | 13 | 1.9 | 13 | 100 | 0 | 50 |
| 156,111 | 156,146 | 18 | 2 | 18 | 100 | 0 | 72 |
| 179,611 | 179,691 | 16 | 1.9 | 16 | 93 | 0 | 53 |
| 192,179 | 192,209 | 12 | 2.6 | 12 | 94 | 0 | 53 |
| 192,228 | 192,324 | 42 | 2.3 | 42 | 92 | 0 | 158 |
| 192,245 | 192,338 | 42 | 2.3 | 40 | 85 | 5 | 118 |
| 213,915 | 213,942 | 11 | 2.5 | 11 | 100 | 0 | 56 |
| 231,980 | 232,022 | 21 | 2.1 | 21 | 95 | 4 | 79 |
| 256,534 | 256,591 | 29 | 2 | 29 | 96 | 0 | 107 |
| 332,270 | 332,314 | 23 | 2 | 23 | 100 | 0 | 90 |
| 352,493 | 352,517 | 13 | 1.9 | 13 | 100 | 0 | 50 |
| 402,386 | 402,421 | 18 | 2 | 18 | 100 | 0 | 72 |
| 449,217 | 449,244 | 11 | 2.5 | 11 | 100 | 0 | 56 |
| 470,821 | 470,913 | 42 | 2.3 | 39 | 80 | 10 | 114 |
| 470,835 | 470,931 | 42 | 2.3 | 42 | 92 | 0 | 158 |
| 470,950 | 470,980 | 12 | 2.6 | 12 | 94 | 0 | 53 |
| 483,468 | 483,498 | 16 | 1.9 | 16 | 93 | 0 | 53 |
| 503,332 | 503,508 | 83 | 2.1 | 82 | 79 | 9 | 191 |
| 537,478 | 537,515 | 19 | 2 | 19 | 100 | 0 | 76 |
| 547,600 | 547,642 | 21 | 2 | 21 | 90 | 0 | 68 |
| 547,695 | 547,722 | 14 | 2 | 14 | 100 | 0 | 56 |
| 571,344 | 571,520 | 83 | 2.1 | 82 | 79 | 9 | 191 |
| 576,088 | 576,112 | 12 | 2.1 | 12 | 100 | 0 | 50 |
| 677,652 | 677,709 | 29 | 2 | 29 | 100 | 0 | 116 |
