# Supplementary material for: The genome and transcriptome of perennial ryegrass mitochondria
Source: BMC Genomics. 2013 Mar 23;14:202. doi: 10.1186/1471-2164-14-202 (PMC3664089; doi:10.1186/1471-2164-14-202)
Supplement: Additional file 4: Table S4 — SSRs found in the perennial ryegrass mitochondrial genome. aPlus and minus, encoded by the forward and reverse DNA strand, respectively; bplus and minus, present and absent, respectively; clower case letter indicates the exon; RC= Reverse complement. [file 1471-2164-14-202-S4.docx]

**Additional file 5: Supplementary Table S4 - Microsatellites found in the mitochondrial genome of perennial ryegrass.**

| Repeat | From  (bp) | To  (bp) | Type | ^a^DNA  strand | ^b^Coding region | ^c^Gene name |
| --- | --- | --- | --- | --- | --- | --- |
| (AAG)^3 | 1,874 | 1,883 | Trinucleotide | + | + | *cox*1 |
| (CTATT)^4 | 2,717 | 2,737 | Pentanucleotide | - | - | - |
| (CATT)^3 | 3,915 | 3,927 | Tetranucleotide | - | - | - |
| (CTT)^3 | 6,650 | 6,659 | Trinucleotide | - | - | - |
| (AAG)^3 | 7,362 | 7,371 | Trinucleotide | + | - | - |
| (AGG)^3 | 16,907 | 16,916 | Trinucleotide | + | - | - |
| (AGT)^3 | 17,137 | 17,146 | Trinucleotide | - | - | - |
| (AAT)^3 | 19,558 | 19,567 | Trinucleotide | + | - | - |
| (GAT)^3 | 19,760 | 19,769 | Trinucleotide | - | - | - |
| (CTT)^3 | 21,355 | 21,364 | Trinucleotide | - | - | - |
| (AAG)^3 | 21,543 | 21,552 | Trinucleotide | + | - | - |
| (CTT)^3 | 22,517 | 22,526 | Trinucleotide | - | - | - |
| (AGT)^3 | 23,417 | 23,426 | Trinucleotide | - | - | - |
| (AGC)^3 | 25,458 | 25,467 | Trinucleotide | + | - | - |
| (ATT)^3 | 26,374 | 26,383 | Trinucleotide | - | - | - |
| (GGT)^3 | 27,891 | 27,900 | Trinucleotide | - | - | - |
| (CTT)^3 | 28,418 | 28,427 | Trinucleotide | - | - | - |
| (ACG)^3 | 36,741 | 36,750 | Trinucleotide | + | - | - |
| (AAG)^3 | 39,018 | 39,027 | Trinucleotide | + | - | - |
| (CTT)^3 | 41,063 | 41,072 | Trinucleotide | - | - | - |
| (CTT)^3 | 41,583 | 41,592 | Trinucleotide | - | - | - |
| (CGT)^3 | 44,501 | 44,510 | Trinucleotide | - | - | - |
| (AGC)^3 | 46,182 | 46,191 | Trinucleotide | + | - | - |
| (GTT)^3 | 46,614 | 46,623 | Trinucleotide | - | - | - |
| (T)^10 | 49,975 | 49,985 | Mononucleotide | - | - | - |
| (A)^10 | 52,188 | 52,198 | Mononucleotide | + | - | - |
| (GCT)^3 | 53,802 | 53,811 | Trinucleotide | - | - | - |
| (GAT)^3 | 55,873 | 55,882 | Trinucleotide | - | - | - |
| (GGT)^5 | 56,023 | 56,038 | Trinucleotide | - | - | - |
| (ACCT)^3 | 61,736 | 61,748 | Tetranucleotide | + | - | - |
| (AAG)^3 | 69,880 | 69,889 | Trinucleotide | + | - | - |
| (AGC)^3 | 73,398 | 73,407 | Trinucleotide | + | - | - |
| (ACT)^3 | 73,986 | 73,995 | Trinucleotide | + | - | - |
| (ATAGT)^3 | 75,187 | 75,202 | Pentanucleotide | - | - | - |
| (GGT)^3 | 76,844 | 76,853 | Trinucleotide | - | - | - |
| (CATT)^3 | 76,974 | 76,986 | Tetranucleotide | - | - | - |
| (AAG)^3 | 81,807 | 81,816 | Trinucleotide | + | - | - |
| (AAG)^3 | 83,056 | 83,065 | Trinucleotide | + | + | *rps*1 |
| (A)^11 | 86,578 | 86,589 | Mononucleotide | + | - | - |
| (CTT)^3 | 86,757 | 86,766 | Trinucleotide | - | - | - |
| (T)^11 | 89,479 | 89,490 | Mononucleotide | - | - | - |
| (T)^12 | 90,264 | 90,276 | Mononucleotide | - | - | - |
| (A)^10 | 91,138 | 91,148 | Mononucleotide | + | - | - |
| (CTT)^3 | 91,898 | 91,907 | Trinucleotide | - | - | - |
| (AGT)^4 | 93,413 | 93,425 | Trinucleotide | - | - | - |
| (AAG)^3 | 93,875 | 93,884 | Trinucleotide | + | + | *rps*4-1 |
| (CTT)^3 | 93,984 | 93,993 | Trinucleotide | - | + | *rps4*-1 |
| (ATC)^3 | 97,184 | 97,193 | Trinucleotide | + | - | - |
| (AAG)^3 | 101,026 | 101,035 | Trinucleotide | + | - | - |
| (AAG)^3 | 103,786 | 103,795 | Trinucleotide | + | - | - |
| (CTT)^3 | 105,668 | 105,677 | Trinucleotide | - | - | - |
| (AAG)^3 | 107,216 | 107,225 | Trinucleotide | + | - | - |
| (AAC)^3 | 107,351 | 107,360 | Trinucleotide | + | - | - |
| (AAG)^3 | 107,394 | 107,403 | Trinucleotide | + | - | - |
| (CTT)^3 | 107,630 | 107,639 | Trinucleotide | - | - | - |
| (AGT)^3 | 107,674 | 107,683 | Trinucleotide | - | - | - |
| (CATT)^3 | 115,600 | 115,612 | Tetranucleotide | - | - | - |
| (CATT)^3 | 115,613 | 115,625 | Tetranucleotide | - | - | - |
| (AGC)^3 | 122,761 | 122,770 | Trinucleotide | + | - | - |
| (ACT)^3 | 127,485 | 127,494 | Trinucleotide | + | - | - |
| (CTT)^3 | 129,478 | 129,487 | Trinucleotide | - | - | - |
| (A)^13 | 129,976 | 129,989 | Mononucleotide | + | - | - |
| (T)^11 | 135,387 | 135,398 | Mononucleotide | - | - | - |
| (CCG)^3 | 140,871 | 140,880 | Trinucleotide | + | - | - |
| (GTT)^3 | 158,864 | 158,873 | Trinucleotide | - | - | - |
| (AAAG)^3 | 160,164 | 160,176 | Tetranucleotide | + | - | - |
| (ACT)^3 | 165,417 | 165,426 | Trinucleotide | + | - | - |
| (ACT)^3 | 166,880 | 166,889 | Trinucleotide | + | - | - |
| (AAG)^3 | 167,678 | 167,687 | Trinucleotide | + | - | - |
| (T)^10 | 172,453 | 172,463 | Mononucleotide | - | - | - |
| (AAG)^3 | 174,608 | 174,617 | Trinucleotide | + | - | - |
| (GAT)^3 | 175,964 | 175,973 | Trinucleotide | - | - | - |
| (AAAC)^3 | 176,409 | 176,421 | Tetranucleotide | + | - | - |
| (ACCAT)^3 | 179,129 | 179,144 | Pentanucleotide | + | - | - |
| (GAT)^3 | 192,225 | 192,234 | Trinucleotide | - | - | - |
| (AAG)^3 | 196,430 | 196,439 | Trinucleotide | + | - | - |
| (AAG)^3 | 196,784 | 196,793 | Trinucleotide | + | - | - |
| (CTT)^3 | 202,179 | 202,188 | Trinucleotide | - | - | - |
| (ACT)^3 | 210,377 | 210,386 | Trinucleotide | + | - | - |
| (AAG)^3 | 212,165 | 212,174 | Trinucleotide | + | - | - |
| (AAG)^3 | 213,502 | 213,511 | Trinucleotide | + | - | - |
| (GCT)^3 | 213,699 | 213,708 | Trinucleotide | - | - | - |
| (ACTG)^3 | 213,761 | 213,773 | Tetranucleotide | + | - | - |
| (CTT)^3 | 215,850 | 215,859 | Trinucleotide | - | - | - |
| (A)^11 | 220,245 | 220,256 | Mononucleotide | + | - | - |
| (AAG)^3 | 223,595 | 223,604 | Trinucleotide | + | - | - |
| (AAG)^3 | 223,881 | 223,890 | Trinucleotide | + | - | - |
| (CATT)^3 | 229,723 | 229,735 | Tetranucleotide | - | + | *rps*3b |
| (A)^10 | 229,768 | 229,778 | Mononucleotide | + | + | *rps*3b |
| (GAT)^3 | 230,720 | 230,729 | Trinucleotide | - | + | *rps*3b |
| (AAC)^3 | 235,240 | 235,249 | Trinucleotide | + | - | - |
| (ACT)^3 | 236,079 | 236,088 | Trinucleotide | + | - | - |
| (ATC)^3 | 238,618 | 238,627 | Trinucleotide | + | - | - |
| (CCT)^3 | 238,788 | 238,797 | Trinucleotide | - | - | - |
| (ACT)^3 | 239,726 | 239,735 | Trinucleotide | + | - | - |
| (ATC)^3 | 246,955 | 246,964 | Trinucleotide | + | - | - |
| ATC)^3 | 248,829 | 248,838 | Trinucleotide | + | - | - |
| (AAG)^3 | 250,102 | 250,111 | Trinucleotide | + | - | - |
| (AAG)^3 | 250,519 | 250,528 | Trinucleotide | + | - | - |
| (AGT)^3 | 252,733 | 252,742 | Trinucleotide | - | - | - |
| (ATT)^3 | 253,999 | 254,008 | Trinucleotide | - | - | - |
| (CTT)^3 | 257,708 | 257,717 | Trinucleotide | - | - | - |
| (T)^11 | 258,053 | 258,064 | Mononucleotide | - | - | - |
| (AAG)^3 | 261,808 | 261,817 | Trinucleotide | + | - | - |
| (ACAT)^3 | 264,165 | 264,177 | Tetranucleotide | + | - | - |
| (AAG)^3 | 264,894 | 264,903 | Trinucleotide | + | - | - |
| (AAG)^3 | 269,358 | 269,367 | Trinucleotide | + | - | - |
| (ATC)^3 | 275,354 | 275,363 | Trinucleotide | + | - | - |
| (T)^10 | 284,852 | 284,862 | Mononucleotide | - | - | - |
| (CTT)^3 | 284,870 | 284,879 | Trinucleotide | - | - | - |
| (CGT)^3 | 285,731 | 285,740 | Trinucleotide | - | - | - |
| (GAT)^3 | 285,865 | 285,874 | Trinucleotide | - | - | - |
| (CCT)^3 | 286,531 | 286,540 | Trinucleotide | - | - | - |
| (AATT)^3 | 287,718 | 287,730 | Tetranucleotide | + | - | - |
| (AAT)^4 | 292,392 | 292,404 | Trinucleotide | + | + | *atp*8 |
| (AAT)^3 | 293,176 | 293,185 | Trinucleotide | + | - | - |
| (ATT)^3 | 294,730 | 294,739 | Trinucleotide | - | - | - |
| (CTT)^3 | 297,811 | 297,820 | Trinucleotide | - | - | - |
| (ACT)^3 | 299,304 | 299,313 | Trinucleotide | + | - | - |
| (AAC)^3 | 300,324 | 300,333 | Trinucleotide | + | - | - |
| (ACT)^3 | 300,582 | 300,591 | Trinucleotide | + | - | - |
| (AGC)^3 | 302,201 | 302,210 | Trinucleotide | + | - | - |
| (T)^10 | 304,760 | 304,770 | Mononucleotide | - | - | - |
| (CTT)^3 | 306,747 | 306,756 | Trinucleotide | - | - | - |
| (ATT)^3 | 310,293 | 310,302 | Trinucleotide | - | - | - |
| (ATT)^3 | 311,803 | 311,812 | Trinucleotide | - | - | - |
| (GCT)^3 | 314,086 | 314,095 | Trinucleotide | - | - | - |
| (A)^11 | 320,287 | 320,298 | Mononucleotide | + | - | - |
| (T)^10 | 321,091 | 321,101 | Mononucleotide | - | - | - |
| (GCT)^3 | 325,097 | 325,106 | Trinucleotide | - | - | - |
| (AGT)^3 | 327,315 | 327,324 | Trinucleotide | - | - | - |
| (CTT)^3 | 329,079 | 329,088 | Trinucleotide | - | - | - |
| (CTT)^3 | 329,579 | 329,588 | Trinucleotide | - | - | - |
| (ATC)^3 | 330,279 | 330,288 | Trinucleotide | + | - | - |
| (AGC)^3 | 333,076 | 333,085 | Trinucleotide | + | - | - |
| (CTT)^3 | 335,087 | 335,096 | Trinucleotide | - | - | - |
| (AAC)^3 | 335,889 | 335,898 | Trinucleotide | + | + | *atp*4 |
| (T)^13 | 338,132 | 338,145 | Mononucleotide | - | - | - |
| (AAG)^3 | 338,634 | 338,643 | Trinucleotide | + | - | - |
| (AGT)^3 | 340,627 | 340,636 | Trinucleotide | - | - | - |
| (GCT)^3 | 345,351 | 345,360 | Trinucleotide | - | - | - |
| (AATG)^3 | 352,493 | 352,505 | Tetranucleotide | + | - | - |
| (AATG)^3 | 352,506 | 352,518 | Tetranucleotide | + | - | - |
| (G)^11 | 359,945 | 359,956 | Mononucleotide | - | - | - |
| (ACT)^3 | 360,444 | 360,453 | Trinucleotide | + | - | - |
| (AAG)^3 | 360,488 | 360,497 | Trinucleotide | + | - | - |
| (CTT)^3 | 360,722 | 360,731 | Trinucleotide | - | - | - |
| (GTT)^3 | 360,765 | 360,774 | Trinucleotide | - | - | - |
| (CTT)^3 | 360,900 | 360,909 | Trinucleotide | - | - | - |
| (CTT)^3 | 365,066 | 365,075 | Trinucleotide | - | - | - |
| (ATT)^3 | 366,015 | 366,024 | Trinucleotide | - | - | - |
| (T)^13 | 369,530 | 369,543 | Mononucleotide | - | - | - |
| (GTT)^3 | 369,831 | 369,840 | Trinucleotide | - | - | - |
| (GTTT)^3 | 372,080 | 372,092 | Tetranucleotide | - | - | - |
| (GTT)^3 | 372,135 | 372,144 | Trinucleotide | - | - | - |
| (GATT)^3 | 372,677 | 372,689 | Tetranucleotide | - | - | - |
| (AGT)^5 | 378,795 | 378,810 | Trinucleotide | - | - | - |
| (GTTT)^3 | 382,113 | 382,125 | Tetranucleotide | - | - | - |
| (ATC)^3 | 382,561 | 382,570 | Trinucleotide | + | - | - |
| (CTT)^3 | 383,917 | 383,926 | Trinucleotide | - | - | - |
| (CTT)^3 | 390,846 | 390,855 | Trinucleotide | - | - | - |
| (AGT)^3 | 391,644 | 391,653 | Trinucleotide | - | - | - |
| (AGT)^3 | 393,107 | 393,116 | Trinucleotide | - | - | - |
| (CTTT)^3 | 398,357 | 398,369 | Tetranucleotide | - | - | - |
| (AAC)^3 | 399,660 | 399,669 | Trinucleotide | + | - | - |
| (AGC)^3 | 416,912 | 416,921 | Trinucleotide | + | - | - |
| (CTT)^3 | 420,720 | 420,729 | Trinucleotide | - | - | - |
| (AGT)^3 | 433,364 | 433,373 | Trinucleotide | - | - | - |
| (CTT)^3 | 439,267 | 439,276 | Trinucleotide | - | - | - |
| (CTT)^3 | 439,553 | 439,562 | Trinucleotide | - | - | - |
| (T)^11 | 442,904 | 442,915 | Mononucleotide | - | - | - |
| (AAG)^3 | 447,301 | 447,310 | Trinucleotide | + | - | - |
| (CAGT)^3 | 449,387 | 449,399 | Tetranucleotide | - | - | - |
| (AGC)^3 | 449,452 | 449,461 | Trinucleotide | + | - | - |
| (CTT)^3 | 449,649 | 449,658 | Trinucleotide | - | - | - |
| (CTT)^3 | 450,986 | 450,995 | Trinucleotide | - | - | - |
| (AGT)^3 | 452,774 | 452,783 | Trinucleotide | - | - | - |
| (AAG)^3 | 460,972 | 460,981 | Trinucleotide | + | - | - |
| (CTT)^3 | 466,367 | 466,376 | Trinucleotide | - | - | - |
| (CTT)^3 | 466,721 | 466,730 | Trinucleotide | - | - | - |
| (ATC)^3 | 470,926 | 470,935 | Trinucleotide | + | - | - |
| (ATGGT)^3 | 484,016 | 484,031 | Pentanucleotide | - | - | - |
| (ACT)^3 | 486,182 | 486,191 | Trinucleotide | + | - | - |
| (CGCT)^3 | 486,966 | 486,978 | Tetranucleotide | - | - | - |
| (CTT)^3 | 489,889 | 489,898 | Trinucleotide | - | - | - |
| (ACT)^3 | 491,085 | 491,094 | Trinucleotide | + | - | - |
| (CTT)^3 | 494,369 | 494,378 | Trinucleotide | - | - | - |
| (CTT)^3 | 496,389 | 496,398 | Trinucleotide | - | - | - |
| (ACG)^3 | 500,267 | 500,276 | Trinucleotide | + | + | *rrn*18-1 |
| (AATG)^3 | 501,263 | 501,275 | Tetranucleotide | + | - | - |
| (AATG)^3 | 507,144 | 507,156 | Tetranucleotide | + | - | - |
| (AAC)^3 | 509,495 | 509,504 | Trinucleotide | + | - | - |
| (CTT)^3 | 509,925 | 509,934 | Trinucleotide | - | - | - |
| (ATT)^3 | 510,486 | 510,495 | Trinucleotide | - | + | *nad*6 |
| (C)^10 | 512,167 | 512,177 | Mononucleotide | + | - | - |
| (A)^10 | 512,399 | 512,409 | Mononucleotide | + | + | *nad*1d |
| (AAT)^3 | 517,379 | 517,388 | Trinucleotide | + | - | - |
| (CTT)^3 | 519,665 | 519,674 | Trinucleotide | - | + | *ccm*FCa |
| (GTT)^3 | 521,467 | 521,476 | Trinucleotide | - | + | *ccm*FCb |
| (ATT)^4 | 522,333 | 522,345 | Trinucleotide | - | - | - |
| (CTT)^3 | 524,092 | 524,101 | Trinucleotide | - | - | - |
| (AAT)^3 | 525,704 | 525,713 | Trinucleotide | + | - | - |
| (AGC)^3 | 526,933 | 526,942 | Trinucleotide | + | - | - |
| (GCT)^3 | 527,091 | 527,100 | Trinucleotide | - | - | - |
| (ATCT)^3 | 530,765 | 530,777 | Tetranucleotide | - | - | - |
| (AAG)^3 | 537,755 | 537,764 | Trinucleotide | + | - | - |
| (ACT)^3 | 539,503 | 539,512 | Trinucleotide | + | - | - |
| (AAG)^3 | 540,763 | 540,772 | Trinucleotide | + | - | - |
| (AAG)^3 | 546,012 | 546,021 | Trinucleotide | + | - | - |
| (AAG)^3 | 555,406 | 555,415 | Trinucleotide | + | - | - |
| (CTT)^3 | 557,561 | 557,570 | Trinucleotide | - | - | - |
| (ATC)^3 | 561,029 | 561,038 | Trinucleotide | + | - | - |
| (GTTT)^3 | 566,622 | 566,634 | Tetranucleotide | - | - | - |
| (ACG)^3 | 568,281 | 568,290 | Trinucleotide | + | + | *rrn*18-2 |
| (AATG)^3 | 569,277 | 569,289 | Tetranucleotide | + | - | - |
| (AATG)^3 | 575,156 | 575,168 | Tetranucleotide | + | - | - |
| (AAG)^3 | 576,205 | 576,214 | Trinucleotide | + | - | - |
| (CCT)^3 | 576,527 | 576,536 | Trinucleotide | - | - | - |
| (ATC)^3 | 578,039 | 578,048 | Trinucleotide | + | - | - |
| (ATT)^3 | 581,178 | 581,187 | Trinucleotide | - | - | - |
| (AAC)^3 | 583,289 | 583,298 | Trinucleotide | + | - | - |
| (GGATT)^3 | 588,489 | 588,504 | Pentanucleotide | - | - | - |
| (GAT)^3 | 593,739 | 593,748 | Trinucleotide | - | - | - |
| (CTT)^3 | 595,991 | 596,000 | Trinucleotide | - | - | - |
| (AAG)^3 | 600,350 | 600,359 | Trinucleotide | + | - | - |
| (AAG)^3 | 609,692 | 609,701 | Trinucleotide | + | - | - |
| (CTT)^4 | 612,262 | 612,274 | Trinucleotide | - | - | - |
| (ATCC)^3 | 615,859 | 615,871 | Tetranucleotide | + | - | - |
| (AGC)^3 | 617,489 | 617,498 | Trinucleotide | + | - | - |
| (AAG)^3 | 622,113 | 622,122 | Trinucleotide | + | - | - |
| (CTT)^3 | 624,110 | 624,119 | Trinucleotide | - | - | - |
| (AAG)^3 | 625,713 | 625,722 | Trinucleotide | + | - | - |
| (ATT)^3 | 627,730 | 627,739 | Trinucleotide | - | - | - |
| (GGT)^3 | 633,316 | 633,325 | Trinucleotide | - | - | - |
| (AGG)^3 | 635,293 | 635,302 | Trinucleotide | + | + | *nad*5b |
| (A)^10 | 637,293 | 637,303 | Mononucleotide | + | - | - |
| (AAG)^3 | 637,549 | 637,558 | Trinucleotide | + | - | - |
| (AGT)^3 | 638,179 | 638,188 | Trinucleotide | - | - | - |
| (GCT)^3 | 638,838 | 638,847 | Trinucleotide | - | - | - |
| (AAG)^3 | 642,379 | 642,388 | Trinucleotide | + | - | - |
| (GCT)^4 | 643,111 | 643,123 | Trinucleotide | - | - | - |
| (AGG)^3 | 646,665 | 646,674 | Trinucleotide | + | - | - |
| (GAT)^3 | 651,806 | 651,815 | Trinucleotide | - | - | - |
| (CTT)^3 | 652,369 | 652,378 | Trinucleotide | - | - | - |
| (AAG)^3 | 654,409 | 654,418 | Trinucleotide | + | - | - |
| (GGT)^3 | 656,277 | 656,286 | Trinucleotide | - | - | - |
| (GCT)^3 | 657,306 | 657,315 | Trinucleotide | - | - | - |
| (GAT)^3 | 658,759 | 658,768 | Trinucleotide | - | - | - |
| (AAG)^3 | 660,772 | 660,781 | Trinucleotide | + | - | - |
| (AGC)^3 | 678,499 | 678,508 | Trinucleotide | + | - | - |
