# Supplementary material for: The genome and transcriptome of perennial ryegrass mitochondria
Source: BMC Genomics. 2013 Mar 23;14:202. doi: 10.1186/1471-2164-14-202 (PMC3664089; doi:10.1186/1471-2164-14-202)
Supplement: Additional file 5: Table S5 — Transposable elements in the perennial ryegrass mitochondrial genome. Transposable elements derived from Triticeae (A) and Poaceae (B). Left and right, position of the transposable element in the mitochondrial genome (From/To indicates start/end of positions of the transposable elements. Orientation: d, direct; c, complementary. Sim indicates value of similarity between 2 aligned fragments; Pos is the ratio of positives to alignment length; Mm:Ts is a ratio of mismatches to transitions in the nucleotide alignment. Score, alignment score obtained from blast. [file 1471-2164-14-202-S5.docx]

**Additional file 6: Supplementary Table S5 – Transposable elements in the perennial ryegrass mitochondrial genome.**

A. Triticeae DNA source

| Left position | | Right position | | Orientation | Repbase library  sequences | Repeat class | Sim | Pos/  Mm:Ts | Score |
| --- | --- | --- | --- | --- | --- | --- | --- | --- | --- |
| From | To | From | To |  |  |  |  |  |  |
| 5,532 | 5,639 | 311 | 417 | d | EnSpm-3_HV | DNA/EnSpm | 0.72 | 14.21 | 402 |
| 20,303 | 20,370 | 6,136 | 6,202 | d | EnSpm3_TM | DNA/EnSpm | 0.74 | 15.55 | 246 |
| 124,411 | 124,545 | 3,528 | 3,671 | d | Gypsy-4-I_TA | LTR/Gypsy | 0.67 | 18.09 | 232 |
| 131,314 | 131,382 | 2,716 | 2,784 | d | ROMANI1_I | LTR/Gypsy | 0.71 | 18.00 | 212 |
| 131,441 | 131,510 | 3,454 | 3,522 | d | Gypsy-4-I_TA | LTR/Gypsy | 0.68 | 14.00 | 237 |
| 242,383 | 242,462 | 6,040 | 6,116 | c | DANIELA_TM_I | LTR/Gypsy | 0.70 | 16.92 | 260 |
| 315,908 | 316,668 | 3,321 | 4,084 | d | Gypsy-4-I_TA | LTR/Gypsy | 0.63 | 19.07 | 751 |
| 316,798 | 316,896 | 3,975 | 4,070 | d | LATIDU2_TM_I | LTR/Gypsy | 0.69 | 20.00 | 253 |
| 317,687 | 317,756 | 4,583 | 4,652 | d | ROMANI1_I | LTR/Gypsy | 0.77 | 16.00 | 362 |
| 336,610 | 336,679 | 3,454 | 3,522 | c | Gypsy-4-I_TA | LTR/Gypsy | 0.68 | 14.00 | 237 |
| 336,738 | 336,806 | 2,716 | 2,784 | c | ROMANI1_I | LTR/Gypsy | 0.70 | 17.27 | 212 |
| 343,575 | 343,709 | 3,528 | 3,671 | c | Gypsy-4-I_TA | LTR/Gypsy | 0.67 | 18.09 | 232 |
| 493,012 | 493,088 | 1,716 | 1,792 | d | TAR2_TA | NonLTR/L1 | 0.75 | 23.75 | 339 |
| 507,664 | 507,697 | 3,238 | 3,271 | c | Copia-4_TA-I | LTR/Copia | 0.91 | 990.00 | 233 |
| 508,478 | 508,526 | 2,880 | 2,928 | c | Copia-7_TA-I | LTR/Copia | 0.77 | 18.33 | 238 |
| 565,294 | 565,441 | 6,257 | 6,377 | c | EnSpm-3_HV | DNA/EnSpm | 0.73 | 17.85 | 265 |
| 592,223 | 592,263 | 4,525 | 4,569 | c | MIUSE1_TM | NonLTR/L1 | 0.83 | 16.66 | 212 |
| 620,494 | 620,666 | 2,636 | 2,820 | d | ROMANI1_I | LTR/Gypsy | 0.65 | 17.93 | 262 |
| 623,353 | 623,700 | 3,481 | 3,860 | d | Gypsy-13_TA-I | LTR/Gypsy | 0.67 | 20.61 | 701 |
| 629,015 | 629,084 | 6,829 | 6,897 | c | Copia-6_TA-I | LTR/Copia | 0.71 | 15.83 | 247 |
| 650,409 | 650,474 | 4,590 | 4,656 | c | ROMANI1_I | LTR/Gypsy | 0.77 | 17.50 | 326 |
| 664,641 | 664,908 | 2,380 | 2,650 | d | WHAM3_TM_I | LTR/Gypsy | 0.67 | 18.53 | 426 |

B. Poaceae DNA source

| Left position | | Right position | | Orientation | Repbase library  sequences | Repeat Class | Sim | Pos  /Mm:Ts | Score |
| --- | --- | --- | --- | --- | --- | --- | --- | --- | --- |
| From | To | From | To |  |  |  |  |  |  |
| 5,532 | 5,639 | 311 | 417 | d | EnSpm-3_HV | DNA/EnSpm | 0.72 | 14.21 | 402 |
| 5,973 | 6,026 | 1,747 | 1,806 | d | Copia-22_SB-I | LTR/Copia | 0.80 | 15.00 | 266 |
| 6,261 | 6,427 | 2,211 | 2,387 | d | Copia3-SB_I | LTR/Copia | 0.71 | 17.91 | 539 |
| 18,891 | 18,939 | 752 | 797 | c | Copia-136_SB-I | LTR/Copia | 0.82 | 35.00 | 228 |
| 20,804 | 20,847 | 2,806 | 2,849 | d | Gypsy-32_BD-I | LTR/Gypsy | 0.79 | 11.25 | 248 |
| 42,687 | 43,100 | 3,916 | 4,322 | d | Copia-11_BD-I | LTR/Copia | 0.64 | 24.36 | 610 |
| 43,236 | 43,376 | 4,617 | 4,765 | d | Copia-76_SB-I | LTR/Copia | 0.74 | 32.22 | 401 |
| 56,135 | 56,211 | 10,517 | 10,590 | d | Helitron-N3_ZM | DNA/Helitron | 0.71 | 14.61 | 222 |
| 89,151 | 89,193 | 5,118 | 5,161 | c | LINE1-11_SBi | NonLTR/L1 | 0.84 | 30.00 | 225 |
| 89,538 | 89,643 | 5,518 | 5,633 | d | Gypsy-138_SBi-I | LTR/Gypsy | 0.76 | 17.69 | 438 |
| 91,028 | 91,082 | 2,324 | 2,378 | c | LINE1-72_SBi | NonLTR/L1 | 0.78 | 40.00 | 250 |
| 91,153 | 91,258 | 4,842 | 4,951 | c | LINE1-12_SBi | NonLTR/L1 | 0.71 | 20.00 | 240 |
| 91,284 | 91,352 | 4,595 | 4,664 | c | LINE1-8_OS | NonLTR/L1 | 0.70 | 13.33 | 252 |
| 117,896 | 118,104 | 4,563 | 4,769 | d | Copia-18_BD-I | LTR/Copia | 0.74 | 17.00 | 862 |
| 124,409 | 124,737 | 2,427 | 2,763 | d | Gypsy-114_ZM-I | LTR/Gypsy | 0.68 | 20.63 | 741 |
| 126,767 | 126,875 | 425 | 554 | d | Copia-33_BD-I | LTR/Copia | 0.75 | 14.00 | 343 |
| 128,916 | 129,101 | 3,589 | 3,779 | d | Gypsy-48_SB-I | LTR/Gypsy | 0.68 | 23.33 | 442 |
| 131,323 | 131,656 | 3,335 | 3,702 | d | Gypsy-108_SB-I | LTR/Gypsy | 0.70 | 19.04 | 620 |
| 134,293 | 134,351 | 1,098 | 1,156 | d | ENSPM2_OS | DNA/EnSpm | 0.82 | 35.00 | 242 |
| 149,515 | 149,615 | 1,253 | 1,353 | c | TOS17 | LTR/Copia | 0.76 | 23.75 | 333 |
| 150,292 | 150,390 | 11,272 | 11,374 | c | Helitron-5_ZM | DNA/Helitron | 0.83 | 14.44 | 588 |
| 166,964 | 167,016 | 4,299 | 4,351 | c | Copia-64_SB-I | LTR/Copia | 0.86 | 11.66 | 366 |
| 174,975 | 175,033 | 250 | 308 | c | MuDR-N1_ZM | DNA/MuDR | 0.76 | 12.72 | 314 |
| 177,194 | 177,257 | 1,363 | 1,426 | d | LINE1-13_ZM | NonLTR/L1 | 0.75 | 17.77 | 248 |
| 181,057 | 181,101 | 260 | 304 | d | FMLN1_SI | NonLTR/L1 | 0.95 | 990.00 | 359 |
| 191,526 | 191,579 | 545 | 598 | d | Gypsy-37_BD-I | LTR/Gypsy | 0.85 | 40.00 | 303 |
| 208,669 | 208,769 | 2,439 | 2,557 | d | TREP20 | NonLTR/L1 | 0.69 | 17.05 | 228 |
| 210,124 | 210,274 | 689 | 845 | c | Gypsy-17_BD-I | LTR/Gypsy | 0.70 | 21.11 | 353 |
| 226,384 | 226,461 | 3,131 | 3,208 | d | OSLINE1_2 | NonLTR/L1 | 0.73 | 16.36 | 279 |
| 226,730 | 227,023 | 1,505 | 1,803 | d | LINE1-68_SBi | NonLTR/L1 | 0.67 | 20.75 | 484 |
| 227,082 | 227,708 | 3,168 | 3,785 | d | LINE1-68_SBi | NonLTR/L1 | 0.74 | 17.83 | 2479 |
| 227,716 | 228,078 | 5,237 | 5,592 | d | LINE1-68_SBi | NonLTR/L1 | 0.67 | 18.88 | 666 |
| 240,694 | 240,752 | 198 | 255 | c | DNA-12N_SBi | DNA | 0.79 | 33.33 | 214 |
| 242,383 | 242,447 | 6,052 | 6,116 | c | DANIELA_TM_I | LTR/Gypsy | 0.72 | 18.00 | 254 |
| 254,097 | 254,137 | 1,730 | 1,770 | c | Gypsy-18_SB-LTR | LTR/Gypsy | 0.82 | 23.33 | 230 |
| 267,115 | 267,182 | 3,653 | 3,720 | c | Gypsy-166_ZM-I | LTR/Gypsy | 0.69 | 16.15 | 257 |
| 272,404 | 272,627 | 4,090 | 4,326 | c | SZ-8_I | LTR/Gypsy | 0.70 | 17.93 | 471 |
| 314,274 | 314,788 | 916 | 1,429 | d | SZ-4_I | LTR/Gypsy | 0.69 | 20.42 | 1299 |
| 315,109 | 315,554 | 1,789 | 2,234 | d | Gypsy-19_BD-I | LTR/Gypsy | 0.66 | 20.50 | 488 |
| 315,713 | 318,484 | 2,334 | 5,117 | d | SZ-4_I | LTR/Gypsy | 0.66 | 20.42 | 4981 |
| 330,022 | 330,089 | 1,363 | 1,433 | d | Gypsy-87_ZM-I | LTR/Gypsy | 0.81 | 20.00 | 342 |
| 336,464 | 336,797 | 3,335 | 3,702 | c | Gypsy-108_SB-I | LTR/Gypsy | 0.70 | 20.00 | 620 |
| 339,019 | 339,204 | 3,589 | 3,779 | c | Gypsy-48_SB-I | LTR/Gypsy | 0.68 | 23.33 | 442 |
| 341,245 | 341,353 | 425 | 554 | c | Copia-33_BD-I | LTR/Copia | 0.76 | 15.00 | 343 |
| 343,383 | 343,711 | 2,427 | 2,763 | c | Gypsy-114_ZM-I | LTR/Gypsy | 0.68 | 20.63 | 741 |
| 350,016 | 350,224 | 4,563 | 4,769 | c | Copia-18_BD-I | LTR/Copia | 0.74 | 17.00 | 862 |
| 367,703 | 367,955 | 1,709 | 1,948 | c | ZhAT7_ZM | DNA/hAT | 0.71 | 19.09 | 596 |
| 371,565 | 371,694 | 5 | 142 | d | Copia-33_BD-I | LTR/Copia | 0.78 | 17.33 | 608 |
| 375,525 | 375,585 | 1,495 | 1,554 | d | Gypsy-51_BD-I | LTR/Gypsy | 0.80 | 15.71 | 334 |
| 376,834 | 376,896 | 3,373 | 3,434 | c | Copia-91_SB-I | LTR/Copia | 0.77 | 16.25 | 290 |
| 381,276 | 381,339 | 1,363 | 1,426 | c | LINE1-13_ZM | NonLTR/L1 | 0.75 | 17.77 | 248 |
| 383,500 | 383,558 | 937 | 995 | c | MuDR-N1_ZM | DNA/MuDR | 0.76 | 12.72 | 314 |
| 391,516 | 391,568 | 4,299 | 4,351 | d | Copia-64_SB-I | LTR/Copia | 0.86 | 11.66 | 366 |
| 408,142 | 408,240 | 11,272 | 11,374 | d | Helitron-5_ZM | DNA/Helitron | 0.83 | 14.44 | 590 |
| 408,917 | 409,017 | 1,253 | 1,353 | d | TOS17 | LTR/Copia | 0.76 | 23.75 | 333 |
| 413,128 | 413,232 | 1,700 | 1,804 | c | LINE1-26_SBi | NonLTR/L1 | 0.63 | 19.00 | 208 |
| 422,257 | 422,301 | 3,555 | 3,599 | c | LINE1-66_SBi | NonLTR/L1 | 0.82 | 26.66 | 248 |
| 425,756 | 426,787 | 4,148 | 5,182 | d | Gypsy-7_HV-I | LTR/Gypsy | 0.68 | 24.23 | 1862 |
| 427,014 | 427,193 | 2,253 | 2,433 | c | LINE1-24_SBi | NonLTR/L1 | 0.68 | 16.00 | 427 |
| 427,194 | 427,339 | 3,414 | 3,559 | c | LINE1-24_SBi | NonLTR/L1 | 0.68 | 17.30 | 442 |
| 435,073 | 435,434 | 5,237 | 5,591 | c | LINE1-68_SBi | NonLTR/L1 | 0.67 | 18.88 | 657 |
| 435,442 | 436,069 | 3,168 | 3,785 | c | LINE1-68_SBi | NonLTR/L1 | 0.74 | 17.73 | 2504 |
| 436,128 | 436,421 | 1,505 | 1,803 | c | LINE1-68_SBi | NonLTR/L1 | 0.67 | 20.75 | 484 |
| 436,690 | 436,767 | 3,131 | 3,208 | c | OSLINE1_2 | NonLTR/L1 | 0.73 | 16.36 | 279 |
| 452,885 | 453,035 | 689 | 845 | d | Gypsy-17_BD-I | LTR/Gypsy | 0.70 | 21.11 | 353 |
| 454,390 | 454,490 | 2,439 | 2,557 | c | TREP20 | NonLTR/L1 | 0.69 | 17.05 | 228 |
| 471,580 | 471,633 | 545 | 598 | c | Gypsy-37_BD-I | LTR/Gypsy | 0.85 | 40.00 | 303 |
| 482,058 | 482,102 | 260 | 304 | c | FMLN1_SI | NonLTR/L1 | 0.95 | 990.00 | 359 |
| 492,983 | 493,219 | 4,145 | 4,385 | d | LINE1-26_SBi | NonLTR/L1 | 0.65 | 19.25 | 435 |
| 507,560 | 509,364 | 1,353 | 3,215 | c | COPIA3-I_OS | LTR/Copia | 0.81 | 18.75 | 9409 |
| 551,971 | 552,451 | 4,159 | 4,646 | d | ATLANTYS-I_OS | LTR/Gypsy | 0.67 | 18.44 | 1177 |
| 552,773 | 552,866 | 2,183 | 2,276 | d | SC-10_I | LTR/Copia | 0.69 | 19.23 | 209 |
| 565,292 | 565,437 | 123 | 235 | d | DNA-12N_SBi | DNA | 0.71 | 15.29 | 201 |
| 579,364 | 579,433 | 1,916 | 1,982 | d | LINE1-60_SBi | NonLTR/L1 | 0.72 | 15.45 | 228 |
| 580,878 | 580,940 | 3,244 | 3,305 | c | SZ-4_I | LTR/Gypsy | 0.77 | 21.66 | 284 |
| 586,352 | 587,296 | 1,363 | 2,293 | c | Copia-73_SB-I | LTR/Copia | 0.75 | 17.19 | 3663 |
| 587,709 | 587,755 | 1,022 | 1,070 | c | RLG_scDEL_1_6-LTR | LTR/Gypsy | 0.83 | 60.00 | 264 |
| 620,378 | 620,679 | 2,094 | 2,415 | d | Gypsy-81_SB-I | LTR/Gypsy | 0.67 | 18.88 | 596 |
| 621,901 | 622,315 | 1,987 | 2,406 | d | SZ-56_I | LTR/Gypsy | 0.69 | 21.15 | 946 |
| 623,357 | 623,639 | 3,065 | 3,379 | d | Gypsy63-ZM_I | LTR/Gypsy | 0.70 | 17.20 | 697 |
| 623,953 | 624,005 | 2,000 | 2,052 | c | Gypsy-95_ZM-I | LTR/Gypsy | 0.79 | 18.33 | 275 |
| 624,520 | 624,587 | 2,853 | 2,920 | c | Gypsy-161_ZM-I | LTR/Gypsy | 0.76 | 26.66 | 292 |
| 624,723 | 624,926 | 4,447 | 4,655 | c | OSR42_I | LTR/Gypsy | 0.68 | 19.63 | 39 |
| 626,059 | 626,111 | 4,203 | 4,255 | d | ENSPM6_OS | DNA/EnSpm | 0.73 | 15.55 | 222 |
| 629,015 | 629,084 | 6,075 | 6,143 | c | Copia2_HV_I | LTR/Copia | 0.72 | 18.00 | 260 |
| 630,167 | 630,233 | 980 | 1,047 | d | LTR-2_SBi | LTR/Gypsy | 0.78 | 33.33 | 260 |
| 633,732 | 633,833 | 4,604 | 4,705 | d | LINE1-50_ZM | NonLTR/L1 | 0.70 | 17.50 | 322 |
| 639,181 | 639,241 | 3,166 | 3,224 | d | LINE1-55_ZM | NonLTR/L1 | 0.78 | 22.00 | 266 |
| 639,415 | 639,713 | 101 | 395 | d | LINE1-37_SBi | NonLTR/L1 | 0.68 | 20.27 | 424 |
| 640,510 | 640,572 | 1,840 | 1,902 | d | Copia-41_BD-I | LTR/Copia | 0.74 | 17.77 | 267 |
| 649,782 | 651,624 | 2,860 | 4,659 | c | Gypsy-27_BD-I | LTR/Gypsy | 0.69 | 19.71 | 4184 |
| 651,663 | 651,794 | 2,232 | 2,362 | c | Gypsy-109_ZM-I | LTR/Gypsy | 0.77 | 16.87 | 608 |
| 662,264 | 662,306 | 122 | 164 | c | Gypsy-17_BD-I | LTR/Gypsy | 0.86 | 15.00 | 281 |
| 664,641 | 664,908 | 2,380 | 2,650 | d | WHAM3_TM_I | LTR/Gypsy | 0.67 | 18.53 | 426 |
| 672,027 | 672,080 | 3,485 | 3,544 | d | HELITRON1_OS | DNA/Helitron | 0.78 | 14.28 | 223 |
