# Supplementary material for: The genome and transcriptome of perennial ryegrass mitochondria
Source: BMC Genomics. 2013 Mar 23;14:202. doi: 10.1186/1471-2164-14-202 (PMC3664089; doi:10.1186/1471-2164-14-202)
Supplement: Additional file 6: Figure 1 (A-E) — Checking of nuclear DNA contamination in isolated perennial ryegrass mtDNA. A-E: Lane 1, 100 bp DNA ladder; 2, F1-30 mtDNA; 3, F1-30 genomic DNA and 4–5, genomic DNA of two other genotypes of perennial ryegrass. DNA amplified by five SSR primer sets, G03_075, G03_044, G05_070, G05_071 [72] and LpSSR006 [73], selected from different linkage groups of perennial ryegrass. [file 1471-2164-14-202-S6.pptx]

## Slide 1
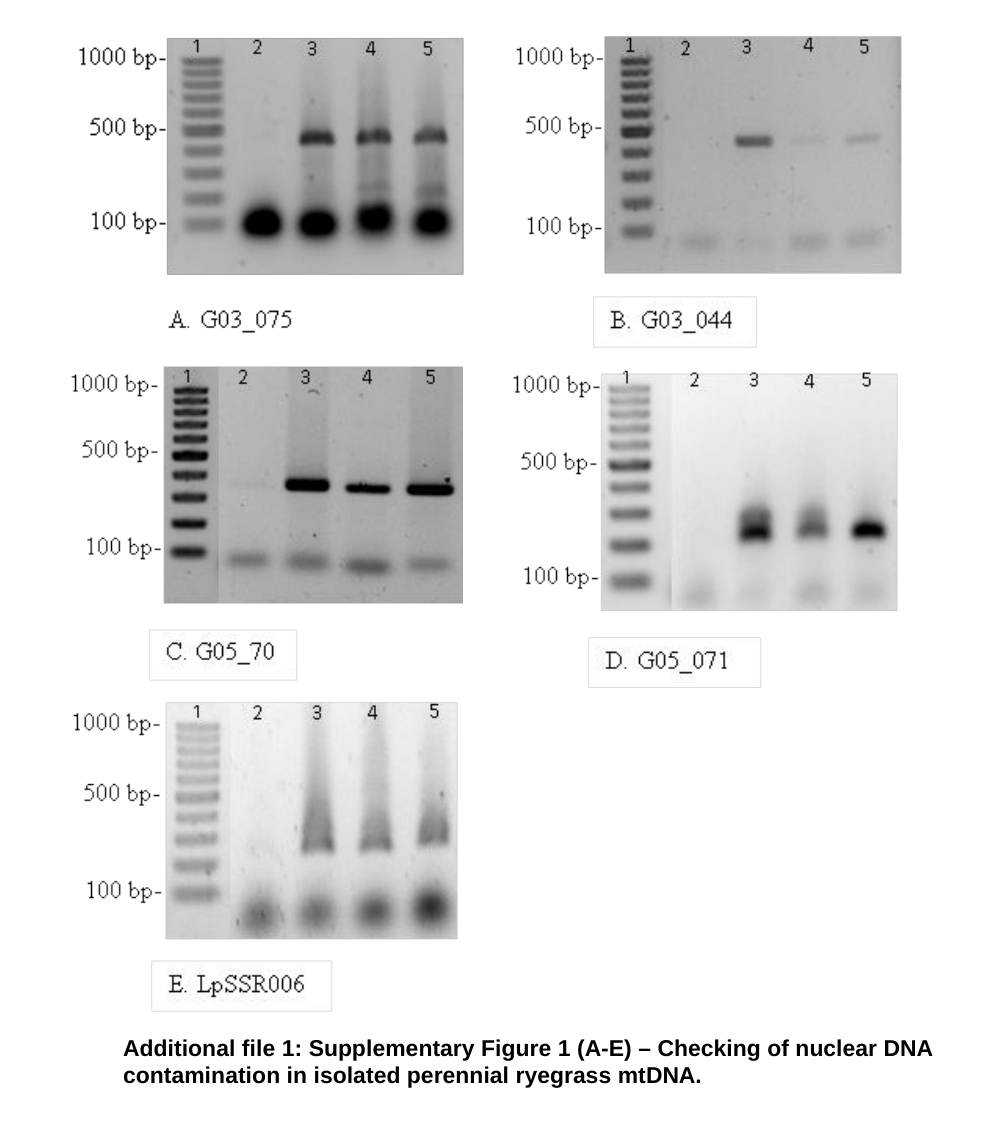

Additional file 1: Supplementary Figure 1 (A-E) – Checking of nuclear DNA contamination in isolated perennial ryegrass mtDNA.
